# Phytic Acid and Its Derivatives as Valuable Flame Retardants for Polymer Systems: Current State of the Art and Perspectives

**DOI:** 10.3390/polym18060671

**Published:** 2026-03-10

**Authors:** Aurelio Bifulco, Giulio Malucelli

**Affiliations:** 1 Department of Chemical, Materials and Industrial Production Engineering (DICMaPI), University of Naples Federico II, Piazzale Tecchio 80, 80125 Napoli, Italy; aurelio.bifulco@unina.it; 2 Department of Applied Science and Technology, Politecnico di Torino, Viale Teresa Michel 5, 15121 Alessandria, Italy; 3 Consorzio Interuniversitario Nazionale per la Scienza e Tecnologia dei Materiali (INSTM), Via G. Giusti 9, 50121 Florence, Italy

**Keywords:** phytic acid, phytates, flame retardancy, textiles, bulk polymers, foams, flammability, combustion behavior

## Abstract

Phytic acid (myo-inositol hexakisphosphate) and its salts, including iron, aluminum, sodium, and lanthanum phytate, are perhaps the most recent discovery in the field of bio-sourced flame retardants. Phytic acid can be extracted from sustainable resources, such as beans, cereals, and oilseeds. Its high phosphorus content (28 wt.% based on molecular weight) organized into six phosphate groups justifies the growing interest this biomolecule has attracted over the last decade in various sectors (as a corrosion inhibitor, antioxidant, and anticancer additive, among others). In addition, when exposed to a flame or an irradiative heat flux, phytic acid is a highly efficient dehydrating and char-forming agent. It also contributes to excellent flame-retardant properties when combined with other carbon sources, such as chitosan, or nitrogen-containing additives, including melamine, urea, and polyethyleneimine. This paper reviews the most recent advances in using phytic acid and its derivatives to design effective flame-retardant systems for textiles, bulk polymers, and foams. It also provides perspectives on possible future developments and implementations.

## 1. Introduction

Flame retardants play a crucial role in improving the fire safety of polymeric materials used in everyday products. However, most polymers are petroleum-based and naturally flammable. The incorporation of flame retardants into the polymer matrix helps to reduce fire risks, slow flame spread and increase the time available for evacuation and emergency response. In general, flame retardants act through different mechanisms able to reduce flame spread, decrease heat release, and limit smoke production during combustion [1,2]. Combustion of polymeric materials is typically a free-radical chain process that involves thermal decomposition (pyrolysis) of the solid, release of flammable volatiles, ignition in the gas phase, and sustained heat feedback from the flame to the material surface. Flame retardants interfere with one or more of these steps through gas-phase chemical action, condensed-phase reactions, physical effects, or combinations thereof. In the gas phase, some flame retardants can disrupt the radical chain reactions responsible for flame propagation. Combustion is sustained by highly reactive radicals such as H^·^ and OH^·^; some flame retardants release species that scavenge these radicals, thereby slowing down or even extinguishing the flame. In the condensed phase, flame retardants may promote the formation of a stable char layer on the material surface. This char acts as a thermal barrier that protects the underlying polymer from heat, reduces the release of flammable volatiles, and limits oxygen diffusion to the polymer surface. Other mechanisms include endothermic decomposition, where flame retardants absorb heat as they decompose (cooling the system), and dilution of combustible gases through the release of inert species such as water vapor, nitrogen or carbon dioxide. Many modern systems combine several of these mechanisms to achieve synergistic performance [3].

The recent restrictions concerning the commercialization of halogen-based flame retardant moved the attention of the scientific community to phosphorus-containing ones, which are particularly versatile because they can act in both the gas and condensed phases, depending on their chemical structure and the polymer matrix [4,5,6]. In the condensed phase, phosphorus compounds often promote char formation by generating phosphoric or polyphosphoric acid derivatives upon thermal decomposition. These acidic species catalyze dehydration and crosslinking reactions in the polymer, especially in oxygen-containing materials like polyesters, epoxies, and cellulose-based polymers. The resulting carbonaceous char layer is thermally stable and can expand (in intumescent systems, thanks to the synergism with nitrogen-based species), leading to the formation of a foamed protective barrier that shields the substrate from heat and oxygen. Intumescent formulations typically combine a phosphorus source (such as ammonium polyphosphate (APP)), a carbon source (char former), and a blowing agent that releases gases to swell the char [7,8,9]. The phosphorus component is essential because it initiates and promotes the dehydration and charring processes that create the protective layer. In the gas phase, certain low-molecular-weight or volatile phosphorus species can enter the flame zone and interfere with radical reactions. Phosphorus-containing radicals, such as PO^·^, are believed to scavenge H^·^ and OH^·^ radicals, thereby reducing flame propagation efficiency. Although this gas-phase effect is generally weaker than that provided by some halogen-based systems, it contributes to overall flame inhibition, especially in systems designed for volatilization [2,3]. An important advantage of phosphorus flame retardants is their adaptability to reactive and additive use. Reactive phosphorus compounds can be chemically bonded into polymer backbones, reducing migration and improving long-term durability, while additive types can be blended into thermoplastics and thermosets [5,6]. The effects of the above flame-retardant mechanisms on the burning of polymeric materials can be effectively investigated by forced-combustion tests (e.g., cone calorimetry measurements), which allow one to estimate important parameters like peak heat release rate (pHRR), total heat release (THR), total smoke release (TSR), total smoke production (TSP), and peak smoke production rate (pSPR) [2].

About 10 to 15 years ago, the academic and industrial flame-retardant community began benefiting from the design and use of bio-sourced products capable of effectively limiting combustion when a direct flame or radiative heat flux is applied to a plastic substrate, such as a bulk polymer system, textile material, or foam. These flame retardants have a low environmental impact and are considered sustainable solutions because they valorize natural materials at the end of their life cycle [10,11,12,13,14,15,16]. Additionally, they typically contain the same active elements, such as nitrogen and/or phosphorus, found in synthetic or fossil-based counterparts; these elements include APP, phosphonates, and polyphosphoric acids. This structural similarity provides comparable flame-retardant behavior in the polymeric materials in which they are embedded or applied [1,6,9,17,18,19,20,21,22,23,24]. From the discovery of the flame-retardant potential of these bio-sourced products, their portfolio has progressively expanded, incorporating polysaccharides, proteins, lignin-rich residues, phenolic compounds, and phosphorus-containing biomolecules [9,25,26,27,28,29,30,31,32,33,34,35,36,37].

**Figure 1:**
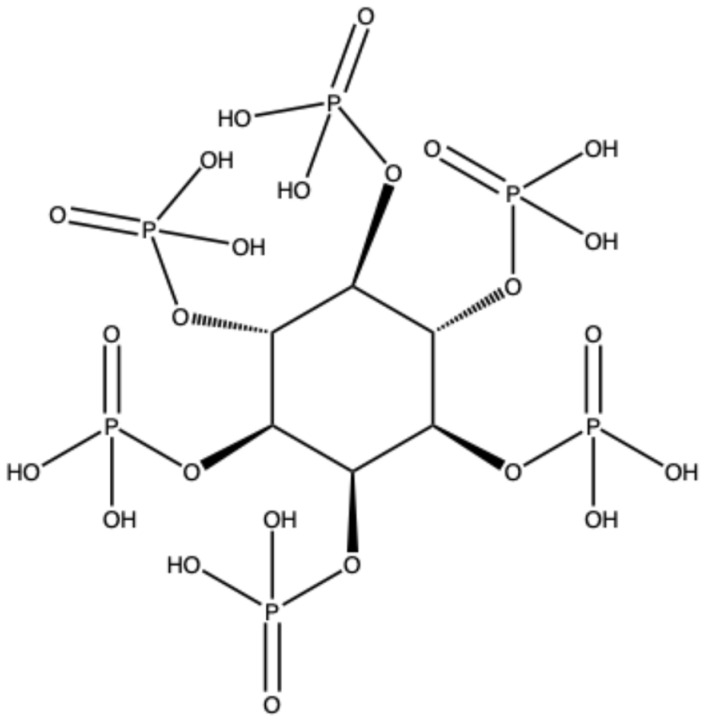
Chemical structure of phytic acid.

Within this broad and rapidly evolving framework, phytic acid (PA) (Figure 1) and its salts have emerged as some of the most promising and versatile bio-derived flame retardants. As discussed in comprehensive analyses of sustainable flame-retardant materials [38] and phosphorus-based polymeric systems [9], the appeal of PA stems from the increasing demand for efficient, low-toxicity, and circular alternatives to conventional flame retardants. PA is naturally abundant in cereals, legumes, and oilseeds. It is characterized by an exceptionally high phosphorus content of around 28 wt.%, which is arranged within six phosphate groups bound to a myo-inositol ring. Apart from its nutraceutical features, this structural motif provides intrinsic dehydrating, charring, and polyphosphate-forming capabilities upon heating. These properties justify the substantial interest that PA has attracted over the last decade [39,40,41,42,43,44].

**Figure 2:**
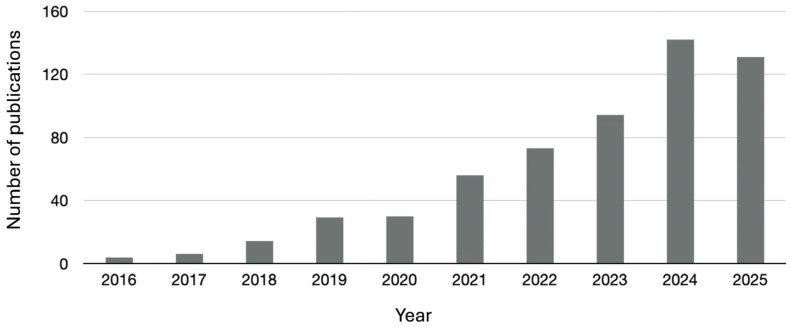
Number of publications (from 2016 to 2025) in peer-reviewed journals, dealing with “Phytic acid AND flame retardancy OR phytates AND flame retardancy” (OR and AND are Boolean operators; data collected from the Web of Science™ database, accessed on 6 November 2025).

The growing academic interest in the use of PA and its derivatives as effective flame retardants is witnessed by the increasing number of articles appearing in the scientific literature (Figure 2).

Recent reviews focusing on PA-based flame retardants for cotton and wool fabrics [45] and biomass-derived PA systems [44] highlight its dual action in both condensed and gas phases. In particular, in the condensed phase, the thermal degradation of PA generates phosphoric and polyphosphoric acids, which catalyze dehydration and carbonization of oxygen-containing substrates, promoting the formation of dense, coherent and highly stable char layers. At the same time, the release of phosphorus-containing radicals in the gas phase extinguishes flame-propagating radicals. This reduces flame intensity and improves fire performance, which is consistent with the behavior of established organophosphorus systems [46,47,48].

Moreover, PA readily interacts with such cationic polymers as chitosan and polyethyleneimine, and with metal ions to form hybrid architectures, including polyelectrolyte complexes and layer-by-layer (LbL) assemblies, as well as a wide family of phytate salts. These interactions produce nanostructured assemblies that impart flame retardancy while retaining the texture, flexibility, and mechanical integrity of textiles [19,49]. Incorporating PA into polymeric substrates, biopolymers, and foams aligns with the ongoing transition toward environmentally friendly flame-retardant methods that use natural acids, amino-rich biopolymers, and nanoparticle-mediated barrier effects [50,51].

Unlike the comprehensive reviews already published in the scientific literature [43,44,45], this review focuses specifically on the most recent advances concerning PA and phytate-based flame-retardant systems. It discusses their chemical reactivity, mechanisms, processing strategies, and emerging applications with a particular focus on 2024 and 2025.

## 2. Phytic Acid and Phytates: General Flame-Retardant Mechanisms

During thermal degradation, PA undergoes dehydration and polycondensation reactions, generating phosphoric and polyphosphoric acid species. These acid intermediates catalyze the dehydration of the polymer matrix or cellulosic substrate, promoting the formation of a thermally stable char layer on the material’s surface. This char layer serves as a physical barrier that strongly limits the heat release and inhibits the diffusion of volatile combustible gases [43,44,45]. In addition, PA decomposes to form water vapor and phosphorus radicals (such as PO· and HPO·), capable of quenching gas-phase combustion radicals, thus interrupting flame propagation [44]. This dual condensed- and gas-phase action mirrors the behavior of advanced phosphorus-based flame retardants such as DOPO and phosphonates [6,52,53,54,55]. PA, therefore, combines several key attributes normally found in different classes of flame retardants, making it unusually multifunctional for a bio-derived molecule.

PA can be directly incorporated into polymeric matrices and textile materials through chemical modification or complexation with metal ions (e.g., Al^3+^, Fe^3+^, and Zn^2+^). Transition-metal phytates, such as iron and nickel phytate (PA-Ni), exhibit strong catalytic charring activity. They promote the formation of dense carbonaceous layers and help suppress smoke release. Aluminum and alkaline earth phytates (e.g., barium phytate) enhance char stability and provide additional thermal buffering, thereby reinforcing the protective barrier formed during combustion. Ammonium phytate contains both phosphorus and nitrogen, a combination that is known to promote intumescence and char expansion [3,56,57,58,59].

These characteristics make PA and phytates highly adaptable to systems that require a tailored thermal response or improved structural integrity of the char residue.

## 3. Flame-Retardant Systems Based on Phytic Acid

The next paragraphs will summarize the main recent outcomes related to the use of PA as an effective flame retardant for bulk polymers, textiles and foams.

### 3.1. Bulk Polymers

#### 3.1.1. Phytic Acid for the Preparation of Nanoparticles and Other Flame-Retardant Systems

Zhang et al. developed hybrids, MoS_2_@Ag@PA, by growing silver nanoparticles on MoS_2_ nanosheets via radiation, then coating them with PA through chelation [60]. Thanks to their lubrication and physical cross-linking, the incorporation of one part of MoS_2_@Ag@PA into EVA (ethylene-vinyl acetate) copolymer boosted melt flow rate by 140% and elongation at break by 17.3%. It also led to a decrease in pHRR, THR, and TSR by 48.7, 42.7, and 41.2%, respectively. In addition, toxic gases like CO were significantly lowered, enhancing fire safety.

Using a similar approach, Xu et al. synthesized silver nanoparticles and modified them with MXene [61]. The name MXene derives from their chemical formula, M_n+1_X_n_T_y_, where M is an early transition metal (e.g., Ti, Zr, Hf, V, or Nb) and X is a carbon or nitrogen. The terminating group T is a crucial part of the chemical formula of most MXene compositions, and commonly refers to chalcogens (O, S, Se, Te) and halides (F, Cl, Br, I). The structure of an MXene sheet is generally characterized by “n + 1” M layers intercalated with “n” X layers and covered with the T groups. Moving back to the work of Xu et al. [61], the resulting material was coated by PA to obtain a core–shell flame retardant (MXene@Ag@PA) for EVA. The incorporation of two parts of MXene@Ag@PA into the copolymer accounted for decreased pHRR (by 30.1%) and TSP (by 28.3%).

Wang et al. prepared a core–shell flame retardant, TiO_2_@LDHs@PANI(polyaniline)-PA, with titanium dioxide as the core and nickel–cobalt layered double hydroxides and phosphorus-doped polyaniline forming the shell [62]. The synthesized flame retardant was incorporated into EVA. The improved flame retardancy of the latter was ascribed to both the carbonization effect (due to the presence of Ni-, Co-, and Ti-based species in the condensed phase) and enhanced compatibility with the EVA matrix [11]. When 10 wt.% of TiO_2_@LDHs@PANI-PA was incorporated into the copolymer, the limiting oxygen index (LOI) of the resulting composite reached 30.2%. In addition, as revealed by vertical flame spread tests, the composite achieved self-extinction and V-0 rating. Moreover, in forced-combustion tests, compared with the unfilled copolymer, pHRR, THR, and pSPR values decreased by about 51, 44, and 62%, respectively.

The peculiar properties of ZIF-67, a zeolitic imidazole framework, which include a large surface area enhancing both porosity and functionality, were exploited by Quan et al. to boost the flame-retardant action of PA-based intumescent species in polypropylene (PP). For this purpose, ZIF-67 was synthesized through a solvent-free reactive extrusion method and incorporated at 5 wt.% loading into the polyolefin, leading to decreases in HRR, TSP, and specific extinction area by 53, 22, and 19%, respectively [63].

To improve the flame retardancy of PP, Lv et al. prepared a hybrid flame retardant (MPA@ZrP-Zn) by noncovalent bonding between MA and PA [64]. The Zr-Zn bimetallic effect was responsible for both the absence of dripping phenomena and the limited smoke emissions during the burning of PP composites containing the hybrid flame retardant. In particular, PP composites with APP (10.5 wt.%), pentaerythritol (5 wt.%), and MPA@ZrP-Zn (at 4.5 wt.% loading) could accomplish UL-94 V-0 rating and an LOI value of 33.5%, together with a significant decrease in THR and pHRR values (by about 15 and 54%, respectively), compared to unfilled PP [64].

Ma et al. functionalized MXene nanosheets by grafting a PA copper complex onto their surface; the so-modified nanofiller was used as flame retardant for the manufacturing of epoxy-based composites [65]. The epoxy matrix demonstrated an increase in its LOI of around 41% when only 2 wt.% modified MXene nanosheets were incorporated into it. In addition, as revealed by forced-combustion tests, the same nanofiller loading accounted for a notable decrease in pHRR (40%) and THR (18%), compared to the unfilled epoxy. The enhanced flame retardancy was ascribed to the charring behavior of MXene and copper irons, combined with the dehydration effected exerted by PA throughout the combustion [65].

Luo et al. combined porous diatomite with melamine (MA) formaldehyde-PA to produce a flame retardant (DMFPA) suitable for epoxy systems [66]. With respect to the unfilled counterpart, epoxy-based composites containing 15 wt.% of flame retardant showed increased residual char (by about 23%) and lower pHRR values (by 56%). In addition, the high porosity of diatomite, the dilution effect exerted by non-flammable gases released during the decomposition of MA, and the inhibition mechanism of PA significantly reduced the smoke factor (by about 78%) with respect to the unfilled epoxy.

Ling et al. exploited a one-step method to develop a flame retardant for epoxy resin, using PA, tannic acid (TA), and polyethyleneimine (PEI) as starting materials [67]. Incorporating 3 wt.% of the flame retardant into the polymer matrix resulted in UL-94 V-0 rating and an LOI value of 28.5%. Due to its charring behavior and the release of biphenyltriol-containing compounds during decomposition, the flame-retardant epoxy resin exhibited lower pHRR (by about 30%), THR (26%), and TSR (21%) compared to its unfilled counterpart [67].

Zuo et al. employed tea polyphenols, epichlorohydrin, L-tryptophan and PA to synthesize an inherent flame-retardant epoxy resin (L-Trp@PA/TPEP) [68]. Compared to pristine solid tea polyphenol-based epoxy monomer (TPEP), L-Trp@PA/TPEP exhibited a remarkable decrease in THR and heat release capacity values (by around 68% and 72%, respectively) in microscale combustion calorimetry tests. It also suppressed dripping phenomena during burning and provided epoxy composites with self-healing features. Finally, the flame-retardant epoxy was used as a coating for lyocell fabrics, providing them with decreased pHRR (by about 91%) and THR (64%) values, compared to the untreated counterpart [68].

Taking inspiration from the good properties of Ti_3_C_2_T_x_ MXene, Gong and co-workers modified UiO-66 (i.e., an archetypal MOF with a very high surface area as well as enhanced thermal stability [69]) with PA and self-assembled the resulting product onto MXene nanosheets, exploiting electrostatic interactions [70]. Incorporating 2 wt.% of the modified nanosheets into an epoxy resin improved the overall thermal and mechanical behavior of the polymer network. Compared to unfilled resin, the composites containing the nanofiller (at 2 wt.% loading) exhibited lower pHRR (by about 32%) and pSPR (36%). These findings were ascribed to the charring behavior of phosphorus-containing acids and transition metal derivatives.

To simultaneously improve the flame retardancy and mechanical performance of epoxy resins, Ji et al. developed a functional additive derived from PA, a covalent organic framework, and Mg/Al-based LDH (layer bimetallic hydroxide), via a solvothermal approach [71]. The presence of only 4 wt.% of the additive provided the epoxy system with UL-94 V-0 rating and 27.1% LOI. The remarkable flame-retardant action of the additive was also witnessed by the significant decrease in pHRR and THR (by about 48% for both), as well as TSP (by around 57%), with respect to unfilled epoxy.

Mo et al. synthesized a novel bio-based flame-retardant polyol (PADEA), reacting PA with diethanolamine [72]. Then, PADEA was reacted in situ with isocyanate to obtain inherently flame-retardant, flexible, and intumescent PU foams. With a PADEA loading of 40 wt.%, the LOI increased by up to 24.1%, which is 22.0% higher than that of flexible PU foams without a flame retardant. Melt dripping phenomena were also significantly reduced in the UL-94 HB tests. Cone calorimetry results showed a 47% decrease in THR. Further characterizations revealed that PADEA could act in both condensed and gas phases.

To confer superior flame retardancy and corrosion resistance to PU composites, Han et al. developed a functional additive by self-assembly of MA, Co^2+^, and PA on the surfaces of α-zirconium phosphate sheets [73]. Then, the so-obtained additive was dispersed in thermoplastic PU. At 5 wt.% of filler loading, the resulting composites showed reduced pHRR, THR, TSP, and total CO production values (respectively by about 36, 18, 16, and 41%), thanks to the formation of a continuous and compact char layer impeding the heat transfer and the release of gaseous products [73].

Zhang et al. exploited vacuum impregnation for preparing flame-retardant shape-stabilized phase change composites, based on a waterborne PU framework, by using MXene in combination with PA dicyandiamide, as filler [74]. While the incorporation of pristine MXene into the polymer matrix gave a maximum phase change temperature of around 37 °C, the exploitation of the modified filler accounted for a significant increase in this temperature up to 45 °C, due to the higher photothermal conversion performance. Compared to pure polyethylene glycol, a typical phase change material, the THR and pHRR values of the modified phase change composites were reduced up to 19 and 36%, respectively [74].

To foster the development of more sustainable solutions, Zhao et al. synthesized a series of bio-based polyols via Friedel–Crafts alkylation, starting from tung oil and salicylaldoxime [75]. These polyols were employed to obtain a tung oil-based PU foam, incorporating a PA-derived flame retardant obtained from the in situ immobilization of iron phenylphosphinate on the surface of PA-activated tung meal-based carbon. The incorporation of 15 wt.% flame retardant into the tung oil-based PU foam allowed for achieving UL-94 V-0 rating, an LOI value of 28.2%, and lower THR (by about 58%) and TSR (70%) values compared to the unfilled material.

Starting with polytrimethylene ether glycol, pentamethylene diisocyanate, and 1,4-butanediol, Yin et al. prepared a fully bio-based thermoplastic PU through a polyaddition reaction; then, PA, modified with (2-aminoethyl)-3-aminopropyltrimethoxysilane (coupling agent), was compounded with PU through melt-blending to obtain environmentally benign flame-retardant composites [76]. The incorporation of PA (at 20 phr loading) into the PU matrix resulted in final composites showing decreased pHRR (by about 56%) and effective combustion heat (24%), compared to unmodified bio-based PU.

Ren and co-workers designed a flame retardant for polyamide 66 by anchoring PEI and PA on the surface of cellulose nanocrystals [77]. The incorporation of the functionalized nanocrystals into polyamide 66 determined an increase in the char yield of around 330%, together with the formation of a compact intumescent char acting as protective barrier during the burning [77].

Ning et al. prepared a polyelectrolyte complex (HTPA) made of N-(2-hydroxyl)-propyl-3-trimethylammonium chitosan chloride and PA and incorporated it into a polyvinyl alcohol (PVA) matrix to prepare composite films [78]. At 1.3 wt.% of HTPA loading, the film exhibited excellent flame retardancy with an LOI of 33.3% and self-extinguishing behavior in vertical flame spread tests. Compared to PVA alone, it also showed 3.4 times greater flexibility and reduced water vapor and oxygen permeability by about 83 and 81%, respectively.

Zhang et al. combined lamellar graphene oxide (GO) and bio-extracted PA with PVA via an eco-friendly water evaporation-induced self-assembly strategy to form a homogeneous composite system [79]. Multiple interactions between PVA, GO, and PA gave rise to a nacre-like cross-linked structure, enhancing tensile strength and toughness over unfilled PVA. Thanks to the barrier effect exerted by GO lamellae, the free radical trapping action deriving from PA and the charring behavior of PA, the obtained composites exhibited a remarkable decrease in pHRR (by about 89%) and THR (66%) compared to the unfilled PVA sample, together with UL-94 V-0 rating and an LOI of 36%. Interestingly, the composites maintained structural integrity after long flame exposure, enabling continuous fire warning for over 2400 s. Fast dehydration and graphitization of composites allowed for a rapid 2 s warning response, with sensitivity to overheating at 150 °C because of the presence of GO, which enhanced thermal accumulation. In addition, when applied as fireproof coatings on wood and cotton, the composites demonstrated excellent flame retardancy and fire-warning ability, showing strong potential for fire protection and alert systems.

To foster the use of PVA in bioplastic and green packaging, Yu et al. employed PVA, dialdehyde starch, and PA to prepare crosslinked polyvinyl alcohol/starch blends, with very low wettability and enhanced flame retardancy [80]. In addition to its flame-retardant properties, PA acted as catalyst for the chemical crosslinking and promoted the establishment of hydrogen bonds between PVA and dialdehyde starch. Interestingly, the optimized formulation containing 15 wt.% of PA accounted for an LOI value as high as 40%, and UL-94 V-0 rating, preventing the dripping of incandescent drops.

Recently, Wu et al. proposed a methodology finalized to obtain flame-retardant PVA films filled by CO_2_ and NH_3_ microbubbles, obtained from the reaction between PA and urea, taking place within the polymer [81]. With respect to the neat polymer, the microbubble-containing films provided PVA with 10% higher LOI values and very low flammability. Song et al. developed a gas-steamed MOF approach to create cheese-like open cages with hierarchical porosity using ZIF-67 as precursor and PA as etchant [82,83]. Acidic steam cleaved the coordination bonds of ZIF-67, forming an open framework with high surface area and complex pores. By incorporating 5 wt.% cages into polyurea (PUA), the final composites exhibited a significant decrease in pHRR, THR, and TSP values by about 38, 26, and 40%, respectively, compared with the unloaded polymer system.

Bansal et al. utilized an LbL method to prepare PA-coated zinc oxide nanoparticles and employed them as efficient flame-retardant additives for PU coatings [84]. The chemical composition of such coatings allowed for combining the flame-retardant effects of PA and ZnO on a nanoscale scaffold. These hybrid nanoparticles could be uniformly dispersed in the PU coatings at 20 wt.% loading, without affecting their physico-mechanical properties, and applied on metal substrates. The coatings embedding the hybrid nanoparticles showed a 50% decrease in flame spread rate, compared to those containing only ZnO. Cone calorimetry tests performed on metal panels coated with PU formulations containing 20 wt.% of PA/ZnO nanoparticles exhibited a significant decrease in pHRR (−25%) and THR (−50%) with respect to the control samples. After combustion, SEM images highlighted the formation of a bubble-like char that could block the diffusion of flammable gases during the burning, demonstrating that PA/ZnO hybrid nanoparticles effectively protect metal substrates from fire.

Exploiting CS-assisted ball milling, making possible the exfoliation of montmorillonite and its co-assembly with PA and urea, Han et al. synthesized a bio-based nano-additive (CPN@MMT) for enhancing the fire performance of PUA composites [85]. CPN@MMT showed very good dispersion in the polymer matrix and, with respect to the unfilled polymer, promoted a significant reduction in pHRR (about 34%), total CO production (40%), and TSP (45%), keeping a stable mechanical response. These findings were ascribed to the formation of a dense and coherent char layer, working as a physical barrier during the burning of the composites.

Yi et al. introduced triglycidyl isocyanurate into the self-assembly of poly(lactic acid) (PLA) and CS to obtain an epoxy-modified bioderived flame retardant (PCT), which could be effectively dispersed in PLA, also exhibiting strong interfacial adhesion with the polymer matrix [86]. The presence of triglycidyl isocyanurate also enhanced the char formation during the burning. Combined with APP modified with an epoxy silane coupling agent (MAPP), the composites containing 2 wt.% of PCT and 1 wt.% of MAPP achieved an LOI of 28.8% and UL-94 V-0 rating, lowering the dripping phenomena of incandescent drops. In addition, the tensile strength of this composite was around 67 MPa, namely 10.8% higher compared to pristine PLA.

Qin et al. flame-retarded PLA by means of a highly effective flame retardant (PTDF), synthesized on purpose through an ionic reaction of PA, terephthalic dihydrazide, and iron salts in water [87]. The incorporation of PTDF into PLA at 4 wt.% loading, together with 0.1 wt.% of polytetrafluoroethylene (as anti-drip additive), provided the composites with UL-94 V-0 grade, as well as an increased LOI (by 5%) compared with unfilled PLA. Interestingly, slightly increasing the PTDF content (6 wt.% loading) in the composites accounted for about 27 and 16% decreases in pHRR and THR, respectively. In addition, the flame retardant acted as a nucleating agent, promoting an increase in the crystallinity of the polymer (by about 26%) and an enhancement of its overall mechanical behavior (tensile strength, maximum impact force, and notch impact strength increased by approximately 12, 37, and 129%, respectively).

Ling et al. modified bamboo activated carbon (i.e., a porous charcoal, typically obtained through a thermo-oxidative treatment—from 600 to 1200 °C—of culms or refuse of mature bamboo plants) with PA, urea, and Zn(NO_3_)_2_·6H_2_O to produce a new carbon-based flame retardant for PLA [88]. PLA composite filled with the tailored carbon-based flame retardant (at 6 wt.% loading) achieved a V-0 rating in UL-94 vertical burning tests and an increased LOI (31.2 vs. 20.1%—unfilled polymer).

Zhong et al. prepared bio-derived multifunctional additives for PLA, employing an aqueous-phase electrostatic self-assembly technique based on the exploitation of PA and nicotinamide as monomers [89]. PLA composites containing only 2 wt.% loading of multifunctional additives exhibited satisfactory mechanical response and could accomplish V-0 rating in UL-94 vertical flame spread tests, also achieving a 29.0% higher LOI than that of unfilled polymer. In addition, the UV protection factor also rose up to 90.8, indicating strong UV shielding performance.

By reacting PA, CS, and resveratrol, Wang et al. synthesized an intumescent flame retardant, for PLA composites [90]. Compared to pure PLA, the composites containing 4 wt.% of the flame retardant reached UL-94 V-0 grade and increased LOI values (26 vs. 19.7%—unfilled polymer); in addition, heat release rate (HRR) was ~21% lower than that of PLA. The intumescent flame retardant also gave a plasticization effect to PLA, facilitating its processing and manufacturing.

Pursuing this research, Gao et al. reacted PA with 2-aminothiazole. The resulting flame-retardant product, incorporated into PLA at 4 wt.% loading, allowed for achieving UL-94 V-0 grade, increased LOI (by about 5.6% with respect to the unfilled polymer), and lowered pHRR values (by about 18%) [91]. The flame retardant was responsible for the formation of a compact char layer limiting the heat exchange at the boundary phase during burning. It also acted as a nucleating additive, increasing the crystallinity of PLA composites by up to 74%.

Lu et al. prepared an efficient flame retardant for PLA through the combination of 3-aminobenzeneboronic acid, CS, PA, and isocyanuric acid triglycidyl ester, using a one-pot method [92]. At 3 wt.% loading, the flame retardant provided PLA with an LOI value of 29.5% and UL-94 V-0 rating, preventing dripping phenomena. The high flame-retardant efficiency of the designed system was attributed to the phosphorus free radicals released by the thermal decomposition of the flame retardant, which behaved as active radical scavengers in the gas phase. The flame retardant was also responsible for an increase in the mechanical strength of the polymer, which achieved about 58 MPa.

With the aim of optimizing the amount of flame retardants needed to improve the fire performance of PLA, Wang et al. synthesized a P/N/S-containing functional material by ionic bonding PA and 2-thiophenethylamine [93]. At 2 wt.% flame-retardant loading, the composites achieved UL-94 V-0 grade and an LOI of 29.3% and showed a strong decrease in HRR and CO_2_ production during forced-combustion tests [93].

Adisa et al. incorporated undoped, doped, and co-doped PANI with several acids, and PANI functionalized with APP or a double-layered hydroxide into polyethylene, through an in situ oxidative polymerization [94]. All the nanocomposites accomplished a UL-94 V-2 rating, except those containing undoped PANI. Compared to unfilled polyethylene, the incorporation of 5 wt.% PANI doped with H_3_PO_4_ into the polymer matrix lowered the pHRR by 20%, which was further reduced by 31% when co-doped PANI with H_3_PO_4_ was incorporated.

To fulfill the growing request of natural substances to enhance fire resistance in elastomer composites, Szadkowski et al. proposed eco-friendly, hybrid flame-retardant additives for natural rubber biocomposites. These additives were obtained through the modification of hydrotalcite and sepiolite with aminosilane and PA [95]. The use of PA-modified fillers (at 10 wt.% loading) reduced the HRR by up to 40%, demonstrating a very good efficiency in flame retardancy and opening the way toward new sustainable strategies to lower the flammability of vulcanized elastomers.

To improve the fire behavior of thermoplastic starch, Liu et al. modified halloysite nanotubes through phosphorylation and blending with PA [96]. Composite films based on thermoplastic starch containing PA-phosphorylated halloysite nanotubes were produced and their overall performance was tested. The incorporation of PA-phosphorylated halloysite nanotubes into the polymer matrix, at 10 wt.% loading, improved the mechanical properties of the latter, while reducing the pHRR, THR, and fire growth index of the thermoplastic starch (by about 66, 21, and 78%, respectively).

Wang et al. prepared a flame-retardant material by inspiration from the architecture of nacre, using cellulose nanofibers as organic component and raw product [97]. More in detail, the nacre-mimetic film materials were obtained by modifying cellulose-rich pulp paper cellulose nanofibers with PA and urea, exploiting a mechanical shearing method. The so-obtained modified cellulose nanofibers were assembled with GO, using CaCl_2_ as a crosslinking agent. Together with a superior tensile strength, the nacre-mimetic films exhibited very low values of pHRR and TSP, which were only around 5% of those of natural wood.

Starting from spruce and hempshives, Antoun et al. fabricated non-flammable binder-free particleboards by hot pressing and steam explosion, using an aqueous extract of PA as flame retardant [98]. Because of swelling phenomena, the flame-retardant treatment with PA negatively impacted the mechanical behavior of the binder-free particleboards. However, the PA-treated materials (at 10 wt.% of PA) exhibited lower average effective heat of combustion values as well as heat release rates, together with longer ignition times.

#### 3.1.2. Phytic Acid for the Manufacturing of Functional Polymeric Coatings

By keeping highly sustainable features in the design strategy, Kang et al. discovered a bio-based intumescent flame-retardant coating (made of hydroxypropyltrimethyl ammonium chloride chitosan, PA, and magnesium gluconate) for PP, rubber, polyurethane (PU) foam, wallpaper, and wood [99]. When heated to 180 °C, the protective film rapidly dehydrated and expanded approximately 137 times, forming a honeycomb char layer. The 200 μm thick coating decreased the HRR of PP by about 64% and increased the time to ignition from 34 to 525 s.

With the aim to enhance the fire performance of epoxy systems and preserve a good mechanical response, Zhong et al. developed a coating made of PA, PANI, and CoFe-Prussian blue analog nanoparticles [100]. The coating was exploited for functionalizing halloysite nanotubes before incorporating them into an epoxy matrix. The so-modified nanotubes were responsible for the formation of a mechanical interlocking structure: in particular, the epoxy system incorporating 5 wt.% nanofiller accounted for a 62.4% increase in the tensile strength compared to the pristine system. In addition, regarding the flame-retardant behavior, the same composite showed an LOI of 33.0% and UL-94 V0 rating, together with reduced values of pHRR (by about 41%) and THR (by 34%) with respect to the unfilled epoxy [100].

To obtain sustainable epoxy coatings with improved fire behavior, Mansouri et al. incorporated PA as a flame retardant into an epoxy resin system crosslinked by bio-based phenalkamine (i.e., a material derived from cashew oil nutshell) [101]. Compared to coatings flame-retarded with a metal oxide and alkyl phosphates, a 10 wt.% loading of PA in the phenalkamine-cross-linked epoxy coatings increased the ignition time and decreased the pHRR and THR by over 25%.

In a further research effort, Lin et al. proposed a PUA coating with flame-retardant and solar de-icing capabilities [102]. Thermochromic microcapsules (TCM) are composed of fatty alcohols, bisphenol A and 2-phenylamino-3-methyl-6-dibutylamino-fluorane, which have high sensitivity in thermal regulation [103]. Using an electrostatic and microencapsulation self-assembly, flame-retardant double-shell microcapsules (TCM@PA/CS-3), made of melamine (MA) resin and PA/chitosan (CS) hybrids, were synthesized and incorporated at 5 wt.% loading into the PUA matrix. Due to the flame-retardant action of PA and the charring behavior of CS, the final composite coatings exhibited a strong delay in the ignition time and around 20% decrease in pHRR. Thanks to the thermochromic effect, the coatings could dynamically adjust their photothermal conversion, promoting the ice melting and slide in 525 s.

Li et al. complexed PA and urea in water to obtain a coating, which was exploited for preparing flame-retardant expandable polystyrene. This latter achieved V-0 UL-94 rating, 42.0% LOI, and excellent smoke suppression [104]. Interestingly, the density of the coated expandable polystyrene increased by 2.3% only with respect to the uncoated counterpart.

Chen et al. obtained a flame-retardant coating for polycarbonate/acrylonitrile butadiene styrene by applying PA and multi-walled carbon nanotubes onto UiO-66, a highly stable, porous MOF made from zirconium clusters and terephthalic acid linkers, via a solvothermal method and incorporating the product into an acrylic resin [105]. At a thickness of around 750 μm, the coating gave a high thermal barrier effect and achieved an LOI value of 27.5%. The condensed-phase action of fillers guaranteed low values of heat release and smoke release throughout the burning of the acrylic resin: these findings were ascribed to the thermal stability of multi-walled carbon nanotubes and char-forming character of UiO-66 [105].

#### 3.1.3. Phytic Acid for the Preparation of Functional Aerogels, Cryogels, and Hydrogels

Using sol–gel chemistry and freeze-drying technology, Wang et al. employed PA and sodium alginate (SA) to fabricate a flame-retardant aerogel, to be employed as a protective coating for rigid polyurethane (RPU) foams [106]. More in detail, the deposition of a 0.5 mm thick aerogel coating on the foam was enough to guarantee a high LOI value (30.4 vol.%) and UL-94 V-0 rating during vertical burning tests. With respect to the uncoated foam, the deposited aerogel accounted for a significant decrease in pHRR, up to about 28%.

In a view of sustainability, Cai et al. obtained a CS-derived composite aerogel film, with radiative cooling and fire-resistance capabilities, through the incorporation of MA/PA hybrids [107]. In particular, the obtained aerogel film was highly reflective and could mask the natural deep-yellow color of CS, conferring to the latter an infrared emissivity of about 90% and a solar reflectivity of ~89%. The 3 mm thick aerogel film could withstand fire exposure (and related temperature of ~500 °C), thanks to the flame-retardant action of MA/PA, inhibiting flame growth and promoting the quick formation of a coherent and protective char.

Pursuing this research, Gong et al. developed biomass intumescent aerogels, based on gelatin, glutaraldehyde, sodium carboxymethyl cellulose, diatomite, and PA, through a freeze-drying method [108]. These aerogels were characterized by low thermal conductivity (between 0.021 and 0.029 W⋅m^−1^⋅K^−1^) and a notable compression modulus of 31.5 MPa. In addition, the aerogels showed an LOI of around 36.5%, UL-94 V-0 rate, and 42% lower TSP values with respect to the unfilled aerogels [108].

**Figure 3:**
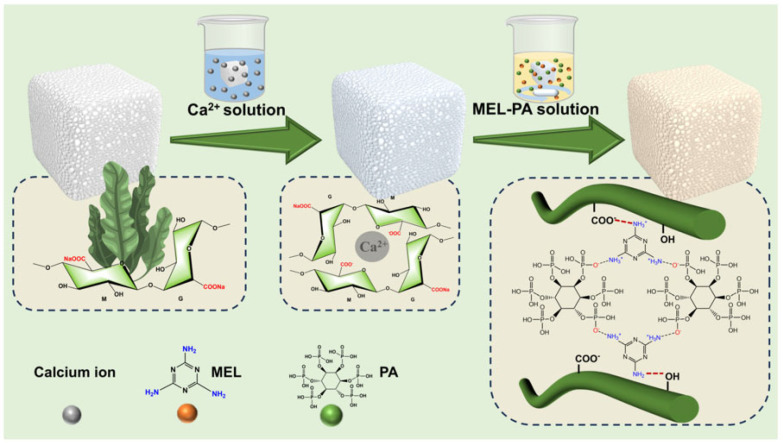
Scheme of the preparation process of composite aerogels. Reprinted from [97] under CC-BY License.

Liu et al. modified an SA aerogel cross-linked with Ca^2+^ with MA and PA to obtain a lightweight and flame-retardant aerogel (Figure 3) [109]. Based on the initial concentrations of MA and PA, the aerogel containing 1.0 wt.% of both flame retardants exhibited V-0 rating in vertical flame spread tests and a notable increase in LOI from 21.5%—unfilled aerogel—to 48.8%.

**Figure 4:**
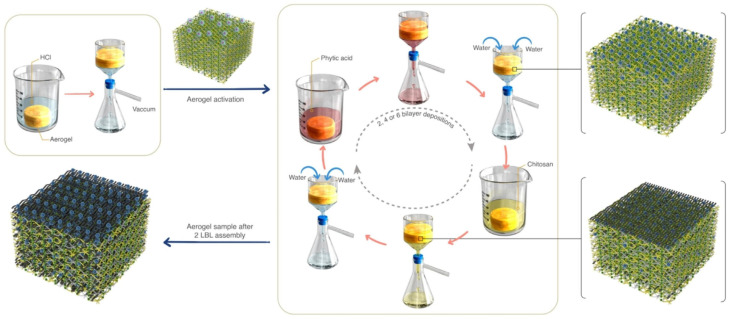
Schematic illustration of the layer-by-layer assembly. By using this method, the original aerogel was modified with depositions of two, four and six bilayers of phytic acid and chitosan. Reprinted from [98] under CC-BY License.

To provide an alternative sustainable material for thermal insulation applications, Varamesh et al. obtained a biobased aerogel system by anchoring on its surface PA and CS through an LbL assembly technique (Figure 4) [110]. The 3D structure of the aerogel was formed by using cellulose filaments and CS, which were crosslinked with citric acid. The aerogel coated with a six-bilayer assembly showed self-extinguishing behavior, together with very low values of pHRR (6.0 kW·m^−2^) and THR (0.4 MJ·m^−2^). Further, this aerogel exhibited a very high LOI value (63%) and superior thermal insulation features, with a thermal conductivity below 38 mW⋅m^−1^⋅K^−1^.

Liu et al. prepared an aerogel composed of hydroxypropylmethyl cellulose and methyltrimethoxysilane, incorporating protonated PEI-PA ligands [111]. The aerogel exhibited 99.24% porosity and very low thermal conductivity. Also, it showed an LOI of 30.2%, together with a significant decrease in pHRR (by about 61%) and THR (58%), compared to unmodified aerogel [111].

Chen et al. developed flame-retardant carbon nanofibers/PANI aerogels with superior flame retardancy and high electromagnetic interference shielding properties [112]. For this purpose, electrospun carbon nanofibers filled by in situ-grown PANI nanoparticles were freeze-dried. The obtained hybrid aerogels exhibited lower pHRR (by about 66%) and THR (by 64%), with respect to their counterparts containing unmodified carbon nanofibers [112].

Wang and co-workers synthesized flame-retardant composite cryogels through the integration of a PA-modified Zr-based metal–organic framework in a CS network [113]. Compared to conventional cryogels based on CS, the cryogels embedding the metal–organic framework at 15 wt.% loading exhibited notable thermal insulating capabilities and decreased pHRR (by 85%) and THR (by 26.3%) with respect to their unfilled counterparts. These cryogels also showed an LOI of 39% and V-0 rating in UL-94 vertical flame spread tests.

Wang et al. exploited facial freeze-casting and freeze-drying for obtaining a biomass porous aerogel, with good fire behavior and excellent thermal insulation features (i.e., long fire response time in direct contact with flame). To this aim, PLA was employed as polymer matrix, and PA and hydroxyapatite as flame retardants [114]. The obtained aerogel achieved UL-94 V-0 rating and showed an LOI of 38.9%. Furthermore, cone calorimetry tests performed on the flame-retarded aerogel revealed decreased THR and TSP values (by 60% and 70%, respectively) compared to the unmodified aerogel. These results suggest that the flame-retarded aerogel is suitable for use in fire alarms.

Xu et al. developed a supramolecular and self-healable hydrogel by compounding PA and TA via dual-nucleophilic esterification in poly(acrylic acid) [115]. The obtained hydrogel was used as protective coating on several polymeric materials: its structure, bearing nanosized pores, could slow down the thermal decomposition and combustion of the underlying polymer, also increasing the time to ignition by more than 2800% and reducing the THR value by 77%.

Li et al. prepared a flame-retardant hydrogel electrolyte made of polyacrylamide (main polymer chain), PA, and pullulan, a natural and water-soluble polysaccharide made of maltotriose and three glucose molecules [116]. The hydrogel, obtained through a one-step radical polymerization route, exhibited a high LOI value (up to 58%).

Pan and co-workers exploited a green method to treat wood aerogel (i.e., wood that was delignified and subsequently freeze-dried) with an intumescent flame-retardant coating made of positively charged PEI and MA and negatively charged PA [117]. The research group demonstrated excellent flame retardancy for the coated wood aerogel, which exhibited self-extinction and decreased pHRR and THR values (respectively, by about 62 and 82%, compared with the uncoated counterpart).

Table 1 provides a summary of the main features and outcomes of the systems described above.

#### 3.1.4. Phytic Acid for the Flame Retardation of Wood-Based Materials

Zhu et al. synthesized an intumescent flame retardant based on PA vanillin arginine salt (VR-PA) by exploiting electrostatic ionic interactions and the Schiff-base reaction, starting from L-arginine, vanillin, and PA [13]. VR-PA was used to obtain fire-resistant wood–PP composites and exhibited notable gas-phase activity during combustion. Compared to composites without the flame retardant, the presence of 20 wt.% VR-PA guaranteed a 28.2% higher LOI and lower pHRR (by about 35%), THR (21%), and TSP (42%) values.

Yang et al. developed a bio-based flame-retardant adhesive. First, they modified microcrystalline cellulose into dialdehyde cellulose. Then, they left it to react with hyperbranched polyamine-melamine via Schiff-base reaction to form a cross-linked polymer [118]. Subsequently, PA was blended with the obtained polymer to create an adhesive, suitable for preparing plywood by hot pressing; the plywood was also coated by a mixture of PA and silica sol–gel derived from tetraethoxysilane. The final coated plywood exhibited an LOI of 38.5% and a THR value reduced by about 89% compared to the uncoated counterpart.

Bao et al. boosted the flame retardancy of undelignified and delignified wood, without affecting its crystalline regions, by impregnation in a solution containing 15 wt.% PA [119]. Subsequently, both woods were dipped into a solution composed of 10 wt.% sodium silicate, 4 wt.% silica nanoparticles, and 4 wt.% aluminum hydroxide. Compared to pristine wood, the fire behavior of the woods was improved, as witnessed by the decrease in THR values by around 47 and 28%, respectively, for delignified and undelignified samples.

To improve its fire behavior and preserve biological durability, Wang et al. treated wood through in situ polyesterification with D-sorbitol and citric acid, catalyzed by PA [120]. Thanks to the flame-retardant action of PA, wood treated with 2 wt.% PA resulted in poor polyester leaching and decreased THR (by about 48%) with respect to pristine wood, and exhibited an LOI of 33.1% [120].

Fan et al. modified 2D mesh-knot metal–organic frameworks with -NH_2_, and further with PA, and employed them to flame-retard wood scaffolds [121]. LOI tests revealed that the frameworks modified with -NH_2_ and PA could increase the LOI value from 22.7 to 30%. Compared to those treated with the frameworks modified with -NH_2_ only, wood scaffolds treated with both -NH_2_ and PA showed lower values of pHRR (by about 56%) and THR (by 69%).

An LbL method was exploited by Liang et al. for fabricating a flame-retardant, antimicrobial, and highly durable coating for wood-based materials. For this purpose, silver nanoparticles were incorporated into a polyhedral oligomeric silsesquioxane (POSS) flame-retardant cage, synthesized by combining 9,10-dihydro-9-oxa-10-phosphaphenathrene (DOPO) with octaminopropyl silsesquioxane; then, the obtained product was LbL-assembled together with CS and PA on wood [122]. The synergy occurring among the components of the LbL architecture led to self-extinction in vertical flame spread tests. In addition, the wood coated with seven layers showed an LOI as high as 30.9%; further, as assessed by forced-combustion tests, the coating was responsible for a significant decrease in HRR and THR values (respectively by about 42 and 44%) compared with the untreated substrate.

Tang et al. developed an LbL self-assembly strategy to produce an intumescent flame-retardant coating for wood pulp paper [123]. This coating was made by interacting negatively charged PA with positively charged polyacrylamide and then functionalized with a polydimethylsiloxane-tetraethoxysilane system. The final wood pulp paper showed an LOI of 35.1%, together with a decrease in THR and pHRR by about 48 and 66%, respectively.

Fire safety of wood can also be significantly improved through the application of organic–inorganic ceramic intumescent coatings. In this context, Yan et al. designed a coating system for wood substrates, by modifying an amino resin with TA and PA and incorporating glass powders or silica [124]. The hybrid component was introduced in the coating through the incorporation of glass powders or silica. With respect to untreated wood samples, the coated ones exhibited a very high LOI (around 49.8%) and reduced THR and TSP (both by about 79%).

Wu and co-workers developed a bio-sourced starch–PA adhesive able to confer highly flame-retardant properties to wood [125]. The research group created a crosslinking network within the adhesive by co-reacting corn starch with PA. Very low loadings of the network based on corn starch (the latter was fixed together with deionized water at 40 g) and filled by PA (20, 30, or 40 g) could increase the wet shear strength of plywood up to 1.09 MPa, fulfilling the requirement of the China National Standard, and lead to an LOI value of about 70% [125].

Fan et al. performed an LbL self-assembly strategy to effectively flame-retard wood [126]. CS, PA, hydrolyzed collagen, and 2D zeolite were systematically deposited on the wood surface to obtain a multifunctional coating. The coated wood samples achieved an LOI of 29.5% and released very little CO during burning. Compared to unmodified wood, samples treated by the multifunctional coating showed a strong decrease in the first pHRR (by about 71%), THR (75%), and second pHRR (53%).

In a further research effort, the same group employed CS, PA, SA, and hydroxyapatite to prepare flame-retardant wood, which was further treated by sodium methyl silicate to lower its wettability and achieve a water contact angle of about 114° [127]. The treated wood achieved self-extinction, an LOI of 34.5%, and very low CO and CO_2_ emissions. In addition, compared to pristine wood, as assessed by forced-combustion tests, the treated wood showed decreased THR and pHRR values (by about 52 and 39%, respectively).

Wei and co-workers used PA, guanazole, and carbon-rich lignin as starting materials for the preparation of a lignin-based intumescent flame retardant [128]. The lignin-based intumescent flame retardant was incorporated into a urea-formaldehyde resin and coated on wood samples. The treated wood achieved a UL-94 V-0 grade in vertical flame spread tests and exhibited an LOI of 36.5%; In addition, compared to uncoated wood, both THR and pHRR values showed a remarkable decrease (respectively by about 93 and 94%).

By employing waste wood as a raw material, Gu et al. exploited a hot-pressing process for preparing advanced composites containing TA and PA as flame retardants, and isocyanate as adhesive [129]. The presence of PA was crucial to achieve superior flame retardancy and smoke suppression. The burning of composites promoted the formation of a huge amount of dense char, enabling for a remarkable decrease in both THR (by around 30%) and TSR (86%).

Shen and co-workers obtained a hydrophobic flame-retardant wood through impregnation of samples with PA and proteolytic product L-arginine [130]. After this impregnation step, SiO_2_ nanoparticles were anchored on the samples’ surface by using polydimethylsiloxane modified with a fluorine-based silane. With respect to untreated wood, the modified counterpart reached self-extinction and UL-94 V-0 rating. In addition, the treatment accounted for a significant decrease in THR and TSP values (by about 49 and 82%, respectively). Finally, the treated wood also showed superhydrophobic features, with water contact angles approaching 156°.

Yan et al. prepared a flame retardant by reacting corn starch with PA; then, the so-obtained product was incorporated into a modified melamine resin to obtain a coating suitable for wood samples [131]. The application of the flame-retardant coating on wood specimens conferred good flame retardancy and smoke suppression effect to the treated materials. In particular, the presence of the flame retardant at 6 wt.% loading in the modified melamine resin accounted for a strong decrease in pHRR (by about 31%), THR (22%), and TSP (48%) values of the coated wood samples: these findings were ascribed to a combined flame-retardant action in both condensed (through extensive charring) and gas (active radical scavenging) phases.

Table 2 provides a summary of the main features and outcomes of the systems based on flame-retardant wood samples.

### 3.2. Phytic Acid in Textile Materials

Shi et al. coated cotton fabrics with dimethyl phosphite lysine (LP) and PA to obtain a high-efficiency fireproof barrier through the establishment of strong electrostatic interaction and hydrogen bonding [132]. The coated fabrics showed an LOI of 40.2% and self-extinction in vertical flame spread tests. In addition, the coating was responsible for a significant decrease in pHRR and THR values (by 74 and 21%, respectively) compared to the reference material, though smoke release increased due to an incomplete combustion. In addition, char residue increased significantly from 7 (neat cotton) to 26%.

Yan et al. applied a simple soaking method to produce a coating composed of PA, L-glutamic acid, and trimesoyl chloride on the surface of cotton fabrics [133]. First, PA and L-glutamic acid were left to interact through electrostatic and hydrogen bonding; then, the stabilization of the coating was achieved via interfacial polymerization involving trimesoyl chloride. The treated fabrics (12 wt.% loading) showed a high LOI of 40%. In vertical flame spread tests, the treated fabrics achieved self-extinction that was maintained even after 300 laundry cycles (the LOI decreased to 30%). Finally, cone calorimetry tests revealed a remarkable decrease in both pHRR and THR compared to untreated cotton (by about 90 and 71%, respectively). The research group also demonstrated that the coating had minimal impact on the mechanical performance of cotton fabrics.

The construction of multilayered polyelectrolyte-based assemblies often requires long procedure times. To overcome this limitation, Wang and co-workers used the step-by-step dip-coating technique for improving the flame retardancy of cotton fabrics [134]. To this aim, PA and CS were self-assembled on the cotton fabric treated with epichlorohydrin-modified aramid nanofibers, an ionic liquid, and a solution of Cu ions. The treated fabric achieved self-extinction in vertical flame spread tests, increasing the LOI value up to 38.5% (vs. 18.5% for untreated cotton). Finally, as assessed by forced-combustion tests, the treated cotton exhibited decreased pHRR and THR values (by about 44 and 55%, respectively).

Wu et al. enhanced the flame retardancy and antibacterial properties of polycotton (i.e., a cheap blend of both cotton and polyester), by applying on its surface a coating made of PA, CS, ferric ion, and polydimethylsiloxane [135]. The coated polycotton exhibited an LOI of 30.2% and a char length of 10.0 cm. The fabric gave over 98% antibacterial activity against *E. coli* and *S. aureus*. The presence of PA in the coating formulation promoted the formation of an intumescent char in the condensed phase, while the release of inert species in the gas phase diluted the fuel load [135].

Safdar et al. used PA combined with 3-(2-aminoethylamino)-propyltrimethoxysilane to obtain flame-retardant coatings for cotton fabrics with durable performance that withstood up to 50 laundry cycles [136]. Further, the coated fabrics exhibited superior thermal stability and achieved an LOI of 31%.

Fang et al. prepared an LbL assembly made of 30 bilayers of GO-PA and carbon nanotubes–CS, and used it to provide cotton fabrics with flame-retardant features [137]. The treated fabrics exhibited an LOI value of 31% and self-extinguishing behavior. The excellent flame retardancy of the treated fabrics was witnessed by the significant decrease in pHRR, THR, and TSP (by about 46, 27, and 82%, respectively), compared to the untreated cotton.

Using a similar approach, Li and co-workers treated cotton fabrics with an LbL assembly made of two bilayers of PEI, PA, and Fe^3+^ or Al^3+^ ions [138]. The treated fabrics were self-extinguishing and achieved an LOI of 35.0% in vertical flame spread tests: these findings were attributed to the formation of a dense char layer inhibiting flame propagation and protecting the underlying fabric [138].

**Figure 5:**
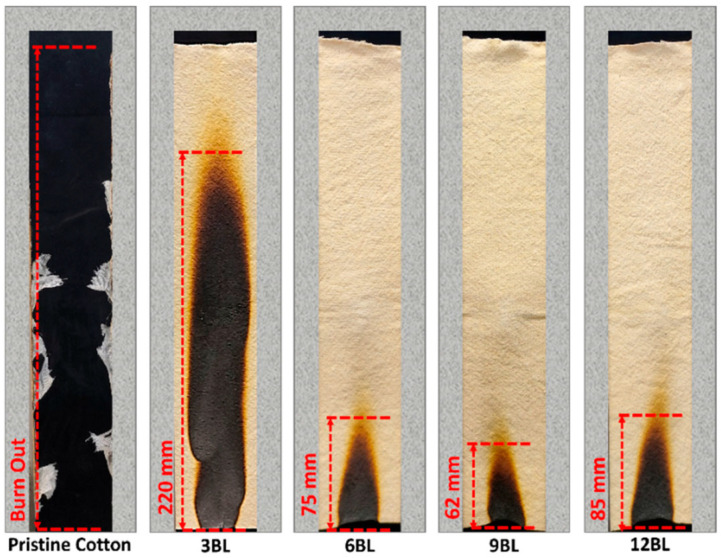
Snapshots taken at the end of vertical flame spread tests carried out on untreated and LbL-treated (*X*BL, where *X* refers to the number of deposited bilayers) cotton fabrics. Reprinted from [139] under CC-BY License.

Yang et al. exploited the LbL method for depositing up to 12 bilayers of pea protein–PA on the surface of cotton fabrics [139]. The combustion of the treated fabrics resulted in the generation of a huge amount of char, thanks to the amino and phosphate groups present in the assemblies, which allowed for achieving self-extinction in vertical flame spread tests, irrespective of the number of deposited bilayers (Figure 5). This char also accounted for a strong decrease in pHRR (25%), THR (54%), and TSP (72%), compared to unmodified cotton fabrics.

Bao et al. developed a multifunctional cotton fabric obtained through impregnation of PA, subsequent in situ growth of zeolitic imidazolate framework-8, and spraying of polydimethylsiloxane [140]. The catalytic charring effect of Zn^2+^ in zeolitic imidazolate framework-8 nanoparticles provided the treated fabrics with an LOI of 34.5%, which only slightly decreased (to 29.6%) after 20 laundry cycles. Compared to the pristine cotton, the treated fabric showed decreased THR and pHRR values, respectively by about 68 and 86%.

Islam et al. improved the fire behavior of cotton fabric by using banana pseudostem sap and PA [141]. The research group applied different concentrations of banana pseudostem sap to pre-mordanted scoured–bleached cotton fabrics, achieving a maximum dry add-on of 4.5 wt.% that accounted for self-extinction in vertical flame spread tests and 27.5% LOI (vs. 18.3% for the untreated cotton).

To provide cotton with durable, antibacterial, and flame-retardant properties, Zhu et al. used PA and N-halamine [142]. Durability tests proved that the antibacterial flame retardant was effectively grafted onto cotton and allowed achieving an LOI value of about 32% self-extinction in vertical flame spread tests, which was kept even after 50 laundry cycles, and an LOI value of about 32%. In addition, as revealed by cone calorimetry tests, the treated cotton exhibited a significant decrease in pHRR (by about 46%) with respect to the reference fabric and a high residue at the end of the tests (around 13%, vs. 0.4% for the untreated cotton). These findings were ascribed to the synergism between PA and N-halamine occurring in the condensed phase with extended charring.

To improve the flame retardancy and the antibacterial properties of lyocell, a regenerated cellulose fiber made from wood pulp through a solvent spinning process, Wang et al. prepared alkaline amino acid-based flame retardants from PA and protein (L-arginine, L-lysine, L-histidine) decomposition products [143]. At ~16.0 wt.% loading, the flame retardant derived from L-arginine gave the best flame-retardant effect, with an LOI as high as 47.1%. Further, cone calorimetry tests revealed that these treated fabrics decreased the pHRR by 92.0% compared with the untreated counterparts. These findings were attributed to flame-retardant mechanisms taking place in both gas and condensed phases. Finally, the lyocell fabrics treated with the flame retardant derived from L-arginine showed antibacterial features, witnessed as a 97.2% inhibition rate against *S. aureus*.

Pursuing this research, the same group applied the same flame retardant derived from L-arginine through double dip–pad–cure processes to lyocell fabrics [144]. The so-treated fabrics achieved an LOI value of 48.3%, which remained high (28.7%) even after 100 laundry cycles. Compared to untreated fabrics, the use of the flame retardant accounted for a remarkable decrease in pHRR and THR (by about 94 and 64%, respectively) in cone calorimetry tests.

Dong et al. modified lyocell fabrics’ surface by using adenosine triphosphate, PA, and dicyandiamide, to obtain good fire response and wearing properties [145]. In particular, the treated fabrics (at 13 wt.% flame-retardant loading) achieved self-extinction in vertical flame spread tests. This formulation exhibited a strong decrease in pHRR (by about 69%) and THR (by 57%), compared to untreated lyocell fabrics. The designed flame-retardant coating provided charring effect, dilution of flammable gases, and flame inhibition during the combustion, without impacting the whiteness and moisture regain features of the fabrics.

Zou and co-workers strongly reduced the flammability of lyocell fabrics by coating them with an electrostatic assembly of PEI, GO, and PA [146]. The deposited coating provided the fabrics with self-extinction; moreover, as assessed by cone calorimetry tests, the treated fabrics exhibited remarkable decreased pHRR and THR values (by about 60 and 48%, respectively), compared to the untreated counterparts.

To improve the flame retardancy of flax fabrics, which are extensively employed in home furnishings, Ishak et al. exploited an e-beam irradiation method for grafting the fabrics with glycidyl methacrylate [147]. Additionally, PA was anchored on the treated fibers to give a phosphorus loading of 0.74 wt.%. As alternative method, PA was also deposed by dip-coating process to increase the phosphorus content up to 1.24 wt.%. While the grafting of glycidyl methacrylate increased the maximum degradation rate, the use of PA favored char formation. Pyrolysis combustion flow calorimetry measurements demonstrated an increase in THR and a lower residue after the grafting of glycidyl methacrylate onto the fabrics. Conversely, the introduction of PA accounted for a significant decrease in THR (by about 42%) and a remarkable increase in TTI (by about 62%), compared with the untreated flax fabric.

Ao et al. proposed a two-step aqueous solution coating method to treat the surface of flax fabrics with PA and CS [148]. The coated substrate (12 wt.% loading) achieved self-extinction in vertical flame spread tests and showed decreased pHRR values (by about 58%) compared with the untreated material.

Yang et al. synthesized a new flame retardant, starting from PA, boric acid, and xylitol to improve the fire resistance of ramie fibers [149]. Then, the treated fabrics were employed for preparing epoxy laminates. Compared to composites containing unmodified fabrics, those with fabrics modified by 6.98 wt.% flame retardant achieved a UL-94 V-0 rating and an LOI value of 31.4%. Meanwhile, THR and TSP decreased by approximately 33 and 31%, respectively.

Kulkarni et al. covalently bonded PA to cotton’s hydroxyl groups of nyco (nylon-cotton) fabric blends to provide the latter with flame-retardant and insect-repellent features [150]. Subsequently, the treated fabrics were coated with an acrylate-based monomer and permethrin to confer insect-repellent capability. The final fabrics showed a 200% increase in char yield upon thermal degradation and achieved self-extinction in vertical flame spread tests, with a char length below 15 cm. Cone calorimetry measurements revealed over 25% reduction in THR, with respect to untreated control.

To design ecofriendly flexible triboelectric nanogenerators from biodegradable natural polymers for self-powered sensing and mechanical-energy harvesting, Yu et al. developed a flame-retardant single-electrode flexible triboelectric nanogenerator by sequentially coating lycra fabrics with conductive polypyrrole and CS/PA [151]. The so-obtained fabrics were self-extinguishing and achieved an LOI of 35.2%: these findings were ascribed to a combined flame retardant action in both condensed (charring) and gas phases (active radical scavenging).

Liu et al. combined PA with guanosine to obtain a fully bio-based flame-retardant coating for PLA fabrics [152]. The flame-retardant coating was also applied on different textile substrates (e.g., cotton, polyester fabrics and films, polyamide fabrics), and accounted for the achievement of self-extinction, preventing the dripping of incandescent drops. In addition, thanks to a dilution effect and inhibition mechanism exerted by the phosphorus-containing radicals in the gas phase, the flame-retardant coating also significantly reduced the heat release of each textile substrate, simultaneously giving rise to the formation of a porous and dense intumescent char layer in the condensed phase.

To limit the detrimental impact on the mechanical properties of PA6 fabrics induced by the strong acidity of PA, Wu et al. improved their fire behavior by compounding PA with ethanolamine [153]. Both chemicals were used to impregnate and coat the surface of polyamide 6 fabrics. Compared to the pristine material, the treated fabrics exhibited 9.2% higher LOI and good mechanical strength retention rate.

Ding et al. developed a flame-retardant hybrid organic–inorganic coating for PET fabrics, using gelatin, PA, and sodium montmorillonite as biobased components [154]. The coating, LbL-assembled on PET, provided self-extinction to the underlying textile material; in addition, as assessed in forced-combustion tests, compared to the untreated fabric, it accounted for a notable decrease in TSR (−63%) and pHRR (−58%), thanks to the formation of an intumescent char containing inorganic species [154].

Ma et al. synthesized a flame-retardant coating made of cellulose nanocrystals, TA, and PA and applied it onto the surface of the polyamide 66 fabric [155]. The coating acted as an effective smoke suppressant, remarkably decreasing TSP and SPR (smoke production rate) values by about 71 and 37%, respectively. TA and PA could improve the fire behavior of the treated polyamide 66 fabric, synergistically exerting a flame-retardant hybrid action in both gas and condensed phases [155].

Table 3 provides a summary of the main features and outcomes of the flame-retardant textile materials described above.

### 3.3. Phytic Acid in Foams

The use of PA in foamed materials was proposed by Wang et al., who fabricated a biodegradable rice straw fiber foam based on APP, PA, and Fe^3+^ ion complexes, via a simple foam molding method [156]. The so-designed complexes, formed on the surface of fabrics (up to 30 wt.% loading), boosted the char production during the burning of the foams. This char, composed of phosphorus-containing carbon layers, was responsible for self-extinction in alcohol lamp tests, as well as for decreased THR and TSR values (both by about 27%) measured on the modified rice straw fiber foam, compared to the reference (i.e., unmodified) system.

In a recent study, Yu et al. prepared a green biomass-derived flame-retardant coating by using PA (as acid source), D-sorbitol (as carbon source), and glycine (as gas source) [157]. The so-obtained coating was employed for providing RPU foams with flame-retardant features. Further, a UV-assisted curing technique was employed to enhance the flame retardancy of the foam substrate. The coated foams achieved 39.7% LOI and accomplished UL-94 V-0 rating in vertical flame spread tests. With respect to uncoated counterpart, the treated foam showed a delay (namely, 3 s) in the ignition time, a 60% decrease in pHRR, and enhanced smoke suppression. The flame-retardant mechanism revealed the formation of a dense char layer during the combustion, along with the release of non-combustible gases [157].

Liu and co-workers prepared a coating made of polyborosiloxane/PA/polyethylenimine, via brush-coating and one-pot foaming techniques [158]. The coating ameliorated the flame retardancy, thermal stability, and heat insulation of the underlying foam. In particular, as assessed by forced-combustion tests, the coated materials exhibited a strong decrease in pHRR (70.1%) and THR (57.0%) values, compared with the untreated counterparts [158].

In conclusion, in comparison to conventional halogenated flame retardants, PA offers several environmental and health advantages, being non-toxic, biodegradable, and derived from renewable agricultural sources. Thanks to its hybrid flame-retardant action in condensed phase (char-forming) and gas phase (radical-trapping via phosphorus-containing volatiles), PA represents an efficient and sustainable alternative for enhancing the fire safety of polymers, textiles, and lignocellulosic composites.

Table 4 provides a summary of the main features and outcomes of the polymeric foams containing phytic acids and its derivatives.

## 4. Flame-Retardant Systems Based on Phytates

The next paragraphs will summarize the main research outcomes related to the use of PA salts as effective flame retardants for bulk polymer, textiles and foams.

### 4.1. Phytates in Bulk Polymers

Duan et al. synthesized quasi-spherical anhydrous magnesium carbonate particles through a deep eutectic solvothermal route and coated them with polydopamine [159]. The product was further functionalized by PA-Ni to obtain a multi-level core–shell flame retardant for EVA copolymers. Due to its charring behavior and inhibition mechanism, the presence of the flame retardant at 50 wt.% led to 30.3% LOI and a UL-94 V-1 rating. In addition, as assessed by forced-combustion tests, pHRR and pSPR values were decreased (respectively by about 63 and 67%) compared with the unfilled copolymer.

Li et al. developed a core–shell flame retardant, APP@PMA–LDH, via a one-pot supramolecular assembly of PA and MA into melamine phytate, which was coated on the surface of APP and loaded with layered double hydroxide (LDH) [160]. The flame retardant was combined with a charring–foaming agent and used as a filler to prepare PP composites. The sample containing 1 wt.% of the core–shell flame retardant showed UL-94 V-0 rating, together with lower pHRR (by about 68%) and TSP (60%) than unfilled PP. These findings were ascribed to the flame retardant that was able to promote the formation of a dense and stable graphitic char on PP’s surface, lowering the release of volatiles and smoke [160].

**Figure 6:**
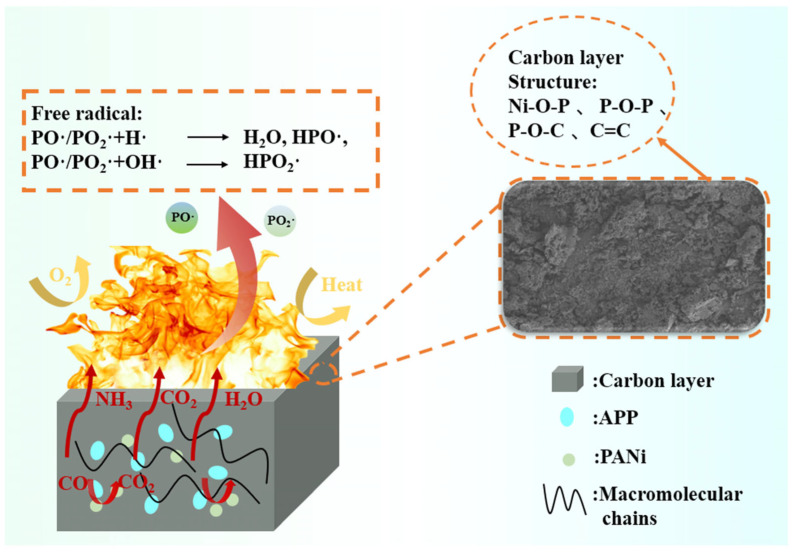
Flame-retardant mechanism of the rigid polyurethane foam incorporating APP and PA-Ni. Reprinted from [156] under CC-BY license.

Zhang et al. synthesized flame-retardant RPU by incorporating APP and self-made PA-Ni into the polymer matrix [161]. With respect to the foam containing PA-Ni alone, the samples filled by 10 wt.% APP achieved 26.5% LOI. In addition, as revealed by cone calorimetry tests, their pHRR and THR were lowered by about 30 and 24%, respectively. These findings were attributed to the occurrence of synergistic effects of APP and PA-Ni in the gas and condensed phases (Figure 6) [161].

**Figure 7:**
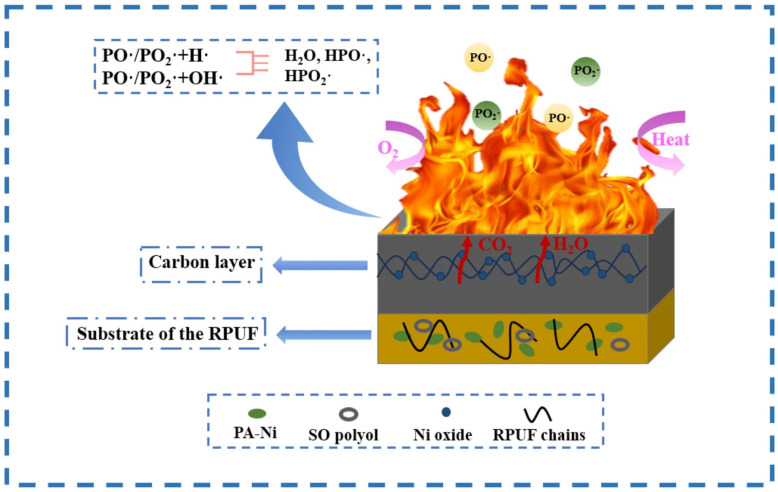
Flame-retardant mechanism of the RPUF incorporating soybean oil-based polyol PA-Ni. Reprinted from [157] under CC-BY license.

In a further research effort to propose a low-carbon RPU foam with enhanced sustainability, the same group developed a flame-retardant material starting from a soybean oil-based polyol and PA-Ni [162]. The foam containing 3 wt.% PA-Ni exhibited 2.6% higher LOI and 14.9% lower pHRR with respect to the unfilled counterpart, together with the lowest specific optical density, under flameless (22.41) or flame (18.90) conditions. Again, the interesting flame retardancy observed in the modified foams was attributed to the PA-Ni action in both condensed (through charring) and gas (through active radical quenching) activities (Figure 7).

**Figure 8:**
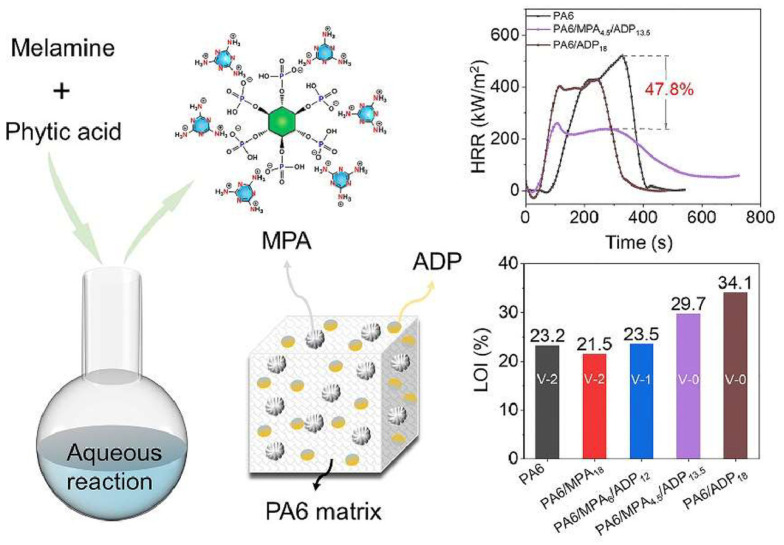
Scheme of the synthesis of the MPA; cone calorimetry curves and LOI values (together with the UL-94 ratings) for unfilled PA6 and its flame-retardant compounds. Legend: PA6/MPAx = PA6 embedding x wt.% of MPA aggregate. PA6/MPAx/ADPy = PA6 embedding x wt.% of MPA aggregate and y wt.% of aluminum diethylphosphinate (ADP). Reprinted from [158] under CC-BY license.

Lu et al. used PA to enhance the fire retardancy of polyamide 6. In particular, a melamine-phytate (MPA) aggregate was prepared via electrostatic interaction and used as a synergist with aluminum diethylphosphinate [163]. Compared to the unfilled polyamide 6, at a 3:1 mass ratio and 18 wt.% total loading, the flame retardant was responsible for a decrease in pHRR, THR, and maximum average HRR by about 48, 27, and 30%, respectively, with a synergistic efficiency of around 43% in pHRR. The flame-retardant polymer achieved a UL-94 V-0 rating and an LOI of 29.7% (Figure 8).

Kong et al. used cellulose nanofibers and PA arginine salt as flame retardants for polybutylene succinate [164]. Blended and bonded cellulose nanofibers and PA arginine salt conferred UL 94 V-0 rating and an LOI value of around 28% to polybutylene succinate-based composites. Compared to pristine polybutylene succinate, 20.5% PA arginine salt and 4.5% cellulose nanofibers blended into polybutylene succinate could provide final products with lower pHRR (by about 45%) compared to the unfilled polymer.

### 4.2. Phytates in Textiles

Kang et al. [165] demonstrated that cellulose fabrics coated with a bio-based, aldehyde-free Zn-phytate/chitosan network exhibit remarkably enhanced flame retardancy, even with an extremely low coating add-on of only 1.5 wt.%. Cone calorimetry tests showed a decrease in pHRR (by about 51%), compared with the untreated fabric. Similarly, THR values decreased by 43%, and the fire growth rate (FIGRA) decreased from 2.1 to 1.2 kW/m^2^/s, indicating a significantly lower fire risk. These results were attributed to four factors, namely (i) active radical quenching by phosphorus species (PO·/HPO·), (ii) catalytic carbonization promoted by Zn^2+^, (iii) formation of a thermally stable Zn–phytate–chitosan char network, and (iv) release of water and fewer hydrocarbons by the treated fabrics during combustion. This resulted in a gaseous atmosphere that was richer in non-combustible species, diluting flammable volatiles and enhancing flame suppression.

Liu et al. [166] incorporated biomass-derived phytate salt into a waterborne PU coating to enhance the flame retardance, anti-drip behavior, and durability of polyester (PET) outdoor fabrics. Adding only 4 wt.% phytate salt enabled the PU-coated PET to self-extinguish in vertical flame spread tests, preventing the dripping of incandescent drops and reducing the damaged length from ~30 cm (untreated fabric) to 12.2 cm. Furthermore, the LOI increased sharply from 21.0% (pristine PET) to 33.8% with a coating add-on of 100 g/m^2^, surpassing commercial flame retardants that usually necessitate higher loadings (8–11 wt.%) for comparable protection. Remarkably, after 50 laundry cycles, the treated fabric retained an LOI as high as 27.8% and remained self-extinguishing. Cone calorimetry tests revealed that pHRR decreased by approximately 29%. Thermogravimetric analyses demonstrated that phytate salt promotes earlier dehydration and increases char residue at the end of tests, indicating a condensed phase flame-retardant mechanism.

Li and co-workers [167] exploited an LbL method to assemble a fully bio-based intumescent coating composed of CS and ammonium phytate. This coating was suitable for providing polyethylene terephthalate (PET) fabrics with enhanced flame retardancy. The treated fabric achieved an LOI of 34.6%, compared to 21.0% for untreated PET. It performed outstandingly in vertical flame spread tests; the damage length decreased from 16 cm for the PET control to 3.4 cm, with zero afterflame, afterglow, and dripping. An expanded, intumescent char layer formed during combustion, which fully covered the fabric, effectively blocking heat and oxygen transfer. Cone calorimetry tests further highlighted the remarkable behavior of the treated fabrics, which showed decreased pHRR and fire growth index (FGI) by about 62 and 82%, respectively. Moreover, the flame-retardant coating acted as an effective smoke suppressant, reducing pSPR by about 48% and limiting the release of CO and CO_2_. These findings were attributed to combined flame-retardant action occurring in the condensed phase (with the formation of highly expanded and stable char) and the gas phase (through a dilution effect). The coating also imparted strong antibacterial activity against *E. coli*, achieving a 99.57% inhibition rate.

Zhou and co-workers [168] designed a multifunctional cotton fabric obtained via a simple two-step spraying method combining ammonium phytate and lignin as a bio-based intumescent flame-retardant layer and PDMS@Fe_2_O_3_-mesoporous silica nanoparticles as a superhydrophobic/UV-shielding overlayer. The flame retardancy was significantly improved: as assessed by vertical flame spread tests, the treated fabrics achieved self-extinction, leaving a char length of only 8.4 cm, compared to complete burn of the pristine cotton. The flame-retardant behavior was attributed to the decomposition of the phytate salt, which gave rise to the formation of pyrophosphoric acid. The latter catalyzed cellulose dehydration and carbonization, forming a dense intumescent char; the overlayer of PDMS@Fe_2_O_3_-mesoporous silica nanoparticles further contributed through the formation of an inorganic (SiO_2_-Fe_2_O_3_) residue acting as thermal shielding. The fabric also showed an outstanding superhydrophobicity, with water contact angles beyond 150°, enabling anti-wetting, anti-fouling, and self-cleaning performance. Importantly, flame retardancy was maintained under water due to the hydrophobic overlayer protecting the underlying flame-retardant layer. Further, the treated cotton showed excellent UV protection, reaching a UPF of 102.18 (50+ rating), with UVA/UVB transmittance below 1%. Finally, the coating exhibited an exceptional durability: after 150 min ultrasonic washing, 50 abrasion cycles, 24 h exposure to strong acid/alkali, and 12 h high-intensity UV irradiation, the flame-retardant fabric retained superhydrophobicity (water contact angles beyond 140°), self-extinguishing ability, and UV protection.

Wu et al. [169] designed and developed a fully bio-based, covalently grafted flame-retardant system for silk fabrics, through the synthesis of vanillin phytate from PA and vanillin and its subsequent grafting onto silk together with diethyl phosphite, exploiting a two-step Kabachnik–Fields reaction. The resulting modified silk (11.5 wt.% loading of the flame retardant) showed an increased LOI (from 23.2%—untreated silk—to 34.7%) and self-extinction in vertical flame spread tests (damaged length: 6.1 cm). Cone calorimetry tests highlighted a remarkable decrease in pHRR and TSP (by about 64 and 90%, respectively). Meanwhile, the char residue increased markedly from 9.7% (untreated silk) to 37.1%, indicating a strong flame-retardant action occurring in the condensed phase. The flame-retardant treatment also showed remarkable durability: in fact, the treated fabric was still self-extinguishing after 25 laundry cycles. Finally, the physical properties (namely, tensile strength, elongation, handle, and whiteness) of silk were only slightly affected by the flame-retardant treatment.

Later, Wu and co-workers employed PA and vanillin to synthesize reactive vanillin phytate ester for tailoring silk fabrics to achieve superior flame retardancy and antibacterial properties [170]. Apart from 97% inhibition rate against *E. coli*, the modified fabrics (22 wt.% of flame-retardant loading) achieved self-extinction in vertical flame spread tests, as well as an LOI value of 33.5%. As assessed by cone calorimetry tests, the flame-retarded fabrics exhibited a remarkable decrease in pHRR and TSR (by about 72 and 40%, respectively), compared to the untreated counterpart. In addition, the proposed treatment showed an interesting durability, as self-extinction was maintained even after 20 laundry cycles.

Liu et al. [171] deposited a bio-based sol–gel coating consisting of γ-ureidopropyltriethoxysilane and ammonium phytate onto cotton fabrics. With a dry add-on of only 9.91 wt.%, the treated textile achieved self-extinction in vertical flame spread tests, reducing the damaged length from 30 cm (control fabric) to 10.1 cm, and raising the LOI from 18.0% to 31.0%. Cone calorimetry tests further confirmed the substantial fire-safety improvement provided by the coating: in particular, pHRR, THR, and TSP values showed a remarkable decrease (by about 48, 48, and 40%, respectively). In addition, the FT-IR analysis of the evolved gases revealed a significant suppression of flammable volatiles and a sustained release of such non-flammable species as H_2_O and CO_2_, confirming an inhibition mechanism taking place in the gas phase. In addition, SEM and Raman analyses of char residues showed the presence of compact, phosphorus- and silicon-rich layers with enhanced graphitization degree, which provided an effective physical barrier against heat and oxygen.

**Figure 9:**
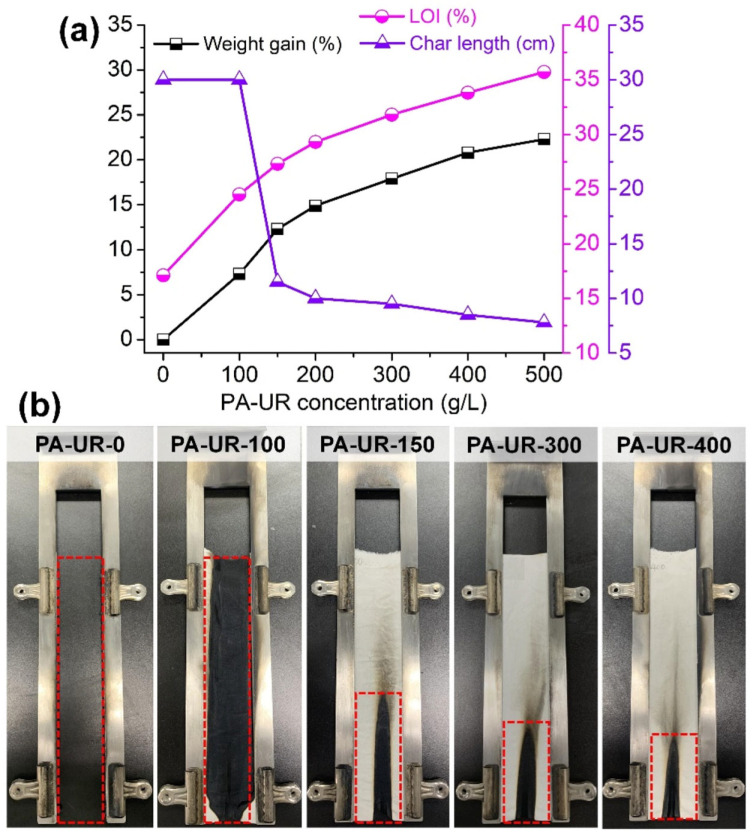
Weight gain, char length, and LOI of polyester/cotton blend fabrics coated with phytic acid-urea salt (**a**) and snapshots after vertical flame spread tests (**b**). Legend: PA-UR-*XXX* = urea-phytate salt, where *XXX* represents the concentration of the salt (in g/L). Reprinted from [139,172] under CC-BY License.

Dong et al. [172] employed a urea-phytate salt coating, synthesized on purpose, for improving the fire performance of polyester/cotton blend fabrics. The treated fabrics markedly enhanced their flame retardancy even at moderate add-on levels (Figure 9a). Indeed, 150 g/L of the urea-phytate was enough to provide the fabric with self-extinction and high LOI values (Figure 9b). Pyrolysis combustion flow calorimetry tests highlighted a decrease in pHRR up to about 42%. These results were ascribed to the formation of P-rich acids and N-containing volatiles during the degradation of the coating, which promote cellulose dehydration, stabilize char, and reduce the available fuel, leading to a synergistic P-N flame-retardant effect in the condensed phase. The only drawback of the proposed flame-retardant coating regarded the limited washing durability: indeed, the treated fabrics lost their flame-retardant behavior after 15 laundry cycles, likely due to insufficient covalent bonding of the coating on polyester-rich substrates.

Cheng et al. [173] synthesized metallic phytate salts, by chelating PA with Mg^2+^, Fe^2+^, Fe^3+^, and Al^3+^; these salts were incorporated into PU coatings to produce durable, flame-retardant outdoor polyester fabrics. Among the tested salts, phytate aluminum salt showed the most stable and efficient flame-retardant behavior without compromising the mechanical performance of the fabric. Unlike PET fabric, which, coated with PU alone, remained highly flammable, burning intensely with severe melt-dripping (damage length = 30 cm, LOI = 20.5%), the incorporation of phytate aluminum salt (at 4 wt.%) into the PU coating yielded a coated PET fabric that self-extinguished, preventing melt dripping phenomena, and increasing the LOI value to 34.2% (a 67% enhancement over uncoated PET). A key outcome was the exceptional washing durability of the treated fabrics: in particular, the fabric treated with the PU coating embedding phytate aluminum salt (at 4 wt.% loading) retained self-extinguishing ability and maintained a high LOI (29.7%) even after 50 laundry cycles. Finally, cone calorimetry tests confirmed a substantial diminution in fire hazard: compared to pristine PET, the treated fabric highlighted a significant decrease in the pHRR, THR, and TSP values (by about 33, 34, and 57%, respectively). These findings were attributed to the phytate aluminum salt, which catalyzed the dehydration and carbonization of the fabric, giving rise to the formation of P-containing acids that accelerate char formation, while Al^3+^ ions promoted the creation of thermally stable inorganic residues, improving structural integrity and heat shielding ability.

Liu and co-workers [174] designed multifunctional cotton fabrics through the application of an eco-friendly coating made of terminated poly(dimethylsiloxane-co-diphenylsiloxane) dihydroxy and ammonia phytate, using a dip-coating process. The so-treated fabrics (10.4 wt.% of dry add-on) achieved self-extinction and LOI values as high as 31.5% (vs. 17.8% of the pristine cotton), thus confirming a strong protection exerted by the coating via phosphate-catalyzed dehydration and carbonization reactions taking place in the condensed phase. Meanwhile, the formation of silica-based structures from the decomposition of the terminated poly-dimethylsiloxane-co-diphenylsiloxane dihydroxy provided a further ceramic barrier behaving as thermal shielding. In addition, as assessed by cone calorimetry tests, the presence of the coating accounted for a significant decrease in the pHRR, THR, and TSP values (by about 89, 51, and 98%, respectively), compared to the control (neat) fabric. Finally, the presence of the terminated poly-dimethylsiloxane-co-diphenylsiloxane dihydroxy in the coating formulation allowed for achieving very high water contact angles (beyond 130°), hence highlighting a strong hydrophobicity of the treated fabrics.

Cheng et al. [175] succeeded in preparing a multifunctional, intumescent coating for polyester/spandex blend fabrics. The coating consisted of carbon black dispersed in a glyceryl phytate ammonium salt, synthesized from PA, glycerol, and urea. The salt acted as a bio-based phosphorus–nitrogen intumescent system, while carbon black enabled char reinforcement, UV shielding, and antistatic functionality. The coated fabrics (dry add-on: 22.6%) achieved self-extinction, prevented melt dripping phenomena, and reached an LOI of 24.2%. Furthermore, as revealed by cone calorimetry tests, the presence of the flame-retardant coating accounted for a significant decrease in pHRR and THR (by about 54 and 40%, respectively), compared to the control fabric. These findings were ascribed to a combined fire-retardant action in condensed (intumescence/charring) and gas (active radical quenching) phases.

Cheng et al. [176] synthesized four bio-based phytate salts through the ionic reaction of PA with polyhexamethylene biguanidine, PEI, melamine, or CS, to be assessed as flame retardants for PU-coated polyamide 6 fabrics. Among them, the flame-retardant salt derived from PA and polyhexamethylene biguanidine showed superior compatibility with PU coatings and the most effective fire-safety performance. As revealed by vertical flame spread tests, unlike untreated polyamide 6 fabrics and the PU-coated counterparts, which burnt vehemently and showed extensive melt-dripping, the fabrics coated with the polyhexamethylene biguanidine-derived phytate (at 4 wt.% loading) achieved self-extinction, and an LOI of 27.7%. In addition, the coating exhibited durability: indeed, after 25 laundry cycles, the coated fabrics were still self-extinguishing. The flame retardancy of these coated fabrics was further confirmed by cone calorimetry tests that highlighted a significant decrease in pHRR, THR, and TSP (by about 39, 22, and 23%, respectively), compared to the control fabric. Again, these results were attributed to a combined flame-retardant action in both condensed (through dehydration and carbonization reactions catalyzed by PA-derived polyphosphates) and gas (dilution effect) phases.

Table 5 provides a summary of the main features and outcomes of the flame-retardant fabrics described above.

### 4.3. Phytates in Foams

Zhang and co-workers [177] proposed barium phytate, synthesized from PA and barium carbonate, as a bio-based, halogen-free flame retardant for flexible PU foams. When incorporated via a one-step foaming process, this phytate substantially enhanced flame resistance, thermal stability, and smoke suppression of the foams. More specifically, cone calorimetry tests allowed for identifying the optimal concentration of barium phytate (i.e., 15 wt.%), which was responsible for the decrease in pHRR and THR values (by about 7 and 29%, respectively), compared with the unmodified foam. These results were ascribed to the decomposition of barium phytate, which gives rise to the formation of pyro- and poly-phosphates, promoting dehydration and intumescence, while Ba^2+^ ions catalyze char formation, yielding a coherent, thermally stable carbonaceous layer that limits volatile release and heat transfer.

Kang et al. [178] proposed melamine-modified zinc phytate as a sustainable flame retardant for silicone rubber foams. In particular, the phytate was prepared via direct precipitation of PA, melamine, and Zn^2+^, yielding a flocculent amorphous composite enriched with P-O, P=O, triazine, and Zn-O functionalities. These chemical motifs enabled an extended charring in the condensed phase, active radical quenching in the gas phase, and metal-catalyzed stabilization of the residual carbon. In particular, the incorporation of 5 wt.% of the phytate into the foam accounted for an LOI value of 29.4% (vs. 21% for the unfilled foam), self-extinction and the achievement of UL-94 V-0 rating. Cone calorimetry tests highlighted a remarkable decrease in pHRR, THR and TSP values (by about 36, 37, and 71%, respectively) compared with the unfilled foams. Once again, these results were attributed to acid-catalyzed dehydration reactions taking place in the condensed phase, as well as to a dilution effect in the gas phase.

### 4.4. Phytates in Wood-Based Materials

As a unique example of applications, Gao et al. used PA and pentaerythritol to prepare pentaerythritol phytate, which was left to interact with hydroxylated boron nitride to obtain a flame retardant able to impregnate densified wood [179]. With respect to the untreated material, the flame-retarded densified wood showed V-0 rating in vertical flame spread tests, as well as an important decrease in peak mass loss rate (by about 57%) and total smoke production (by about 39%) in forced combustion tests.

Table 6 provides a summary of the main features and outcomes of the flame-retardant polymeric systems described above.

## 5. Conclusions and Perspectives

This work has critically reviewed the most recent advances in using phytic acid and its derivatives as bio-based flame retardants for polymers, textiles, foams, and lignocellulosic materials. Due to its exceptionally high phosphorus content, renewable origin, low toxicity, and multifunctional chemical structure, phytic acid has emerged (and is emerging) as an attractive alternative to conventional halogenated and fossil-based flame retardants. The surveyed literature demonstrates that phytic acid and phytates effectively act through combined condensed-phase and gas-phase mechanisms. These mechanisms include char formation, catalytic dehydration, radical quenching, and smoke suppression across a wide range of substrates.

The versatility of phytic acid chemistry allows it to be integrated into materials through various strategies, such as direct incorporation, surface coating, layer-by-layer deposition, ionic complexation, and formation of hybrid organic–inorganic architectures. This adaptability has led to significant improvements in key fire performance parameters, such as LOI, HRR, THR, and smoke production, often at relatively low additive loadings. Furthermore, synergistic effects with nitrogen-containing compounds, metal ions, nanofillers, and biopolymers enhance flame-retardant efficiency while preserving or improving mechanical, barrier, and functional properties.

From a sustainability perspective, phytic acid-based systems align well with current regulatory demands and societal expectations for safer, more environmentally friendly fire-retardant solutions. Their bio-sourced nature, biodegradability, and reduced environmental footprint give them a clear advantage over many standard/conventional flame retardants. In particular, the growing body of research on durable, multifunctional coatings for textiles and wood highlights the potential of phytic acid derivatives to provide flame retardancy and additional features, such as antibacterial activity, UV shielding, (super)hydrophobicity, and thermal insulation.

Despite these promising outcomes, several challenges must be addressed before large-scale industrial implementation can be achieved. These include improving the long-term durability of phytic acid-based treatments following laundry treatment, weathering, and mechanical stress, mitigating possible drawbacks related to acidity and compatibility with certain polymer matrices, and optimizing processing routes to ensure scalability, cost-effectiveness, and reproducibility. In addition, a deeper understanding of structure–property–performance relationships, particularly at the molecular and interfacial levels, is required to rationally and effectively design next-generation phytic acid-derived flame retardants.

Looking ahead, future research should focus on developing reactive and covalently bonded phytic acid systems, advanced hybrid architectures with controlled morphology, and fully bio-based intumescent formulations tailored to specific applications. Also, greater emphasis should be placed on life-cycle assessment, fire toxicity, and real-fire performance to facilitate the transition from laboratory-scale studies to practical, market-ready solutions.

Overall, phytic acid and its derivatives undoubtedly represent key building blocks in the design of sustainable, high-performance flame-retardant materials and are expected to play an increasingly important role in the evolution of environmentally responsible, fire-safe material technologies.

## Figures and Tables

**Table 1:** The flame retardancy performance of flame-retardant polymeric systems incorporating phytic acid and other additives.

Polymeric System	Flame-Retardant Additives and Strategies	Fire Performance (in Comparison with Pristine System)	Ref.
Polyurethane coatings	Phytic acid-coated zinc oxide nanoparticles	25% lower pHRR 50% drop in THR	[84]
Polyvinyl alcohol-based films	Polyelectrolyte complex of N-(2-hydroxyl) -propyl-3-trimethylammonium chitosan chloride and phytic acid	LOI of 33.3% self-extinguishing behavior	[78]
Polyvinyl alcohol/starch bioplastics	Phytic acid and dialdehyde starch	LOI value of 40.0% UL-94 V-0 rating without any dripping	[80]
Polyurea coating	Double-shell microcapsules, made of melamine resin and phytic acid/chitosan hybrids	Strong delay in the ignition time Reduction in pHRR (by 20%)	[102]
Ethylene vinyl acetate copolymer	Hybrids made by growing silver nanoparticles on 2D molybdenum disulfide; subsequent coating of the hybrids with phytic acid	Decrease in pHRR, THR, and TSR (by 48, 43, and 41%, respectively)	[60]
Ethylene vinyl acetate copolymer	Silver nanoparticles modified with MXene, then coating them with phytic acid	Decrease in pHRR (by 30%) and TSP (28%)	[61]
Poly(lactic acid)	Structures based on phytic acid, terephthalic dihydrazide, and iron salts	UL-94 V-0 grade 5% higher LOI than that of pure PLA Decrease in pHRR (by 27%) and THR (16%)	[87]
Poly(lactic acid)	Intumescent filler based on phytic acid, chitosan, and resveratrol	UL-94 V-0 grade Increased LOI values from 19.7 to 26.0% ~21% lower HRR	[90]
Poly(lactic acid)	A system based on phytic acid and 2-aminothiazole	UL-94 V-0 grade 5.6% higher LOI 18% lower pHRR	[91]
Polypropylene	ZIF-67 and phytic acid	Reduction in HRR, TSP, and SEA (by 53, 22, and 19%, respectively)	[63]
Aerogel composed of (hydroxypropyl)methyl cellulose-methyltrimethoxysilane	Protonated polyethyleneimine- phytic acid ligands	LOI of 30.2% Reduction in pHRR (by 61%) and THR (by 58%)	[111]
Poly(lactic acid)-based aerogel	Phytic acid and hydroxyapatite	UL-94 V-0 rating LOI of 38.9% Reduction in THR (by 60%) and in TSP (by 70%)	[114]
Epoxy resin	Halloysite nanotubes functionalized with a coating made of phytic acid, polyaniline, and CoFe-Prussian blue analog nanoparticles	LOI of 33.0% UL-94 V0 rating Reduction in pHRR (by 41%) and THR (by 34%)	[100]
Epoxy resin	Diatomite with melamine formaldehyde-phytic acid	22.7 wt.% higher residual char Decreased pHRR (by 56%) 77.7% lower smoke factor	[66]
Epoxy resin	Phytic acid-modified UiO-66 self-assembled onto MXene nanosheets	Lower pHRR (by 32%) and pSPR (by 36%)	[70]

**Table 2:** The flame retardancy performance of wood treated by phytic acid and other additives.

Polymeric System	Flame-Retardant Additives and Strategies	Fire Performance (in Comparison with Pristine System)	Ref.
Wood	Coating made of amino resin with tannic acid and phytic acid, containing glass powders or silica	LOI of around 49.8% Reduction in THR and TSP (both by 79%)	[124]
Wood	Coating made of chitosan, phytic acid, hydrolyzed collagen, and 2D zeolite	LOI of 29.5% Decrease in first pHRR (by 71%), in THR (by 75%), and in second pHRR (by 53%)	[126]
Wood	Coating made of melamine resin containing corn starch and phytic acid	Decrease in pHRR (by 31%) and in TSP (by 48%)	[131]

**Table 3:** The flame retardancy performance of textiles treated by phytic acid and other additives.

Polymeric System	Flame-Retardant Additives and Strategies	Fire Performance (in Comparison with Pristine System)	Ref.
Cotton fabric	Coating made of phosphite lysine and phytic acid	LOI of 40.2% Self-extinguishing capability Reduction in pHRR by 74% and THR by 21%	[132]
Cotton fabric	Coating treatment based on phytic acid, chitosan, epichlorohydrin-modified aramid nanofibers, ionic liquid, and a solution of Cu ions	LOI value increased from 18.5% to 38.5% Decrease in pHRR (by 44%) and THR (by 55%)	[134]
Cotton fabric	Layer-by-layer assembly composite coating made of carbon nanotubes, graphene oxide, chitosan, and phytic acid	LOI value of 31% Self-extinguishing behavior Reduction in pHRR (by 46%), THR (30%), and TSR (82%)	[137]
Cotton fabric	Impregnation with in situ growth of zeolitic imidazolate framework-8, phytic acid, and spraying of polydimethylsiloxane	LOI of 34.5% Reduction in THR (by 68%) and pHRR (by 86%)	[140]
Cotton fabric	Treatment based on phytic acid and N-halamine	Self-extinguishing behavior In UL-94 tests, damaged length of 67 mm LOI value of 32.2%	[142]
Lyocell fabrics	Amino acid flame retardants from phytic acid and protein decomposition products (arginine, lysine, histidine)	LOI value of 47.1% Decrease in pHRR by 92.0%	[143]
Lyocell fabrics	Phytic acid and L-arginine	LOI value of 48.3% Lower pHRR (by 94%) and THR (by 64%)	[144]
Flax fabrics	Coating made of phytic acid and chitosan, obtained from polysaccharides	Self-extinguishing response Decreased pHRR (by 58%) 200% higher burn-through time	[148]
Polyamide 6 fabric	Coating made of phytic acid and ethanolamine	9.2% higher LOI	[153]

**Table 4:** The flame retardancy performance of foams based on phytic acid and other additives.

Polymeric System	Flame-Retardant Additives and Strategies	Fire Performance (in Comparison with Pristine System)	Ref.
Rigid polyurethane foam	Phytic acid, D-sorbitol, and glycine	LOI of 39.7% UL-94 V-0 rating 60%pHRR decrease	[157]
Rigid polyurethane foam composites	Coating made of polyborosiloxane/ phytic acid/polyethylenimine	A decrease in pHRR (70%) and THR (57%)	[158]
Polyurethane foam	In situ immobilization of iron phenylphosphinate on the surface of phytic acid activated tung meal-based carbon	UL-94 V-0 rating LOI value of 28.2 vol.% Lower (58%) THR and (70%) TSR	[75]
Rigid polyurethane foam	Aerogel based on phytic acid and sodium alginate	LOI value of 30.4 vol.% V-0 rating in UL-94 tests 28% decrease in pHRR	[106]

**Table 5:** The flame retardancy performance of fabrics treated by phytate and other additives.

Polymeric System	Flame-Retardant Additives and Strategies	Fire Performance (in Comparison with Pristine System)	Ref.
Polyester fabrics	Biomass-derived phytate salt into a waterborne polyurethane coating	LOI increased from 21.0% (pristine PET) to 33.8% After 50 washing cycles, the treated fabric retained a high LOI of 27.8% and remained self-extinguishing pHRR decreased by ~29%	[166]
Silk fabric	Vanillin phytate	LOI increased from 23.2% (untreated silk) to 34.7% Self-extinction in vertical flame spread tests Remarkable decrease in pHRR and TSP (by about 64 and 90%, respectively)	[169]
Cotton fabrics	Eco-friendly coating made of poly(dimethylsiloxane-co-diphenylsiloxane) dihydroxy terminated and ammonia phytate	Self-extinction LOI values as high as 31.5% (vs. 17.8% of the pristine cotton) Decrease in pHRR, THR, and TSP values (by about 89, 51, and 98%, respectively)	[174]
Polyamide 6 fabrics	Four bio-based phytate salts through the ionic reaction of phytic acid with polyhexamethylene biguanidine, polyethyleneimine, melamine, or chitosan	Fabrics coated with the polyhexamethylene biguanidine-derived phytate achieved self-extinction, and a LOI of 27.7% After 25 laundry cycles, the coated fabrics were still self-extinguishing Decrease in pHRR, THR, and TSP (by about 39, 22, and 23%, respectively)	[176]

**Table 6:** The flame retardancy performance of polymeric systems based on phytate and other additives.

Polymeric System	Flame-Retardant Additives and Strategies	Fire Performance (in Comparison with Pristine System)	Ref.
Polyamide 6	Melamine-phytate aggregate and aluminum diethylphosphinate	Decreased pHRR (by ~48%), THR (~27%), and MARHE (~30%) UL-94 V-0 rating LOI of 29.7%	[163]
Polypropylene	Melamine phytate coated on the surface of ammonium polyphosphate and loaded with layered double hydroxide	UL-94 V-0 rating Decreased pHRR (by 68%) and TSP (by 60%)	[160]
Rigid poly urethane foam	Soybean oil-based polyol and nickel phytate	2.6% higher LOI Decreased pHRR (by about 15%)	[162]
Flexible polyurethane foams	Barium phytate	Decrease in pHRR and THR values (by about 7 and 29%, respectively) Ba^2+^ ions catalyze char formation, yielding a coherent, thermally stable carbonaceous layer that limits volatile release and heat transfer	[177]

## Data Availability

No new data were created or analyzed in this study.

## Abbreviations

The following abbreviations are used in this manuscript:

APP	Ammonium polyphosphate
EVA	Ethylene vinyl acetate
FGI	Fire growth index
FIGRA	Fire growth rate
GO	Graphene oxide
LbL	Layer-by-layer
LDHs	Layered double hydroxides
LOI	Limiting oxygen index
LP	Dimethyl phosphite lysine
MA	Melamine
MARHE	Maximum average rate of heat emission
MPA	Melamine-phytate
MOFs	Metal–organic frameworks
PA-Ni	PA-Ni
PA	Phytic acid
PANI	Polyaniline
PEI	Polyethyleneimine
PET	Polyethylene terephthalate
pHRR	Peak heat release rate
PLA	Poly(lactic acid)
POSS	Polyhedral oligomeric silsesquioxane
PP	Polypropylene
pSPR	Peak smoke production rate
PU	Polyurethane
PUA	Polyurea
PUF	Polyurethane foam
PVA	Polyvinyl alcohol
RPUF	Rigid polyurethane foam
SA	Sodium alginate
SPR	Smoke production rate
TA	Tannic acid
THR	Total heat release
TSP	Total smoke production
TSR	Total smoke release
WFPP	Wood flour polypropylene
ZIF-67	Zeolitic Imidazole Framework-67
